# Evaluation of amoxicillin and benzylpenicillin therapy in early-onset neonatal sepsis: a pharmacometric external validation and simulation study

**DOI:** 10.1093/jac/dkaf191

**Published:** 2025-06-16

**Authors:** Tom C Zwart, Dimitra Eleftheriou, Sophie J Jansen, Martha T van der Beek, Dirk Jan A R Moes, Swantje Völler, Vincent Bekker

**Affiliations:** Department of Clinical Pharmacy and Toxicology, Leiden University Medical Center, Leiden, The Netherlands; Department of Clinical Pharmacy, Haga Teaching Hospital, The Hague, The Netherlands; Division of Systems Pharmacology and Pharmacy, Leiden Academic Centre for Drug Research, Leiden University, Leiden, The Netherlands; Division of Neonatology, Department of Pediatrics, Willem Alexander Children’s Hospital, Leiden University Medical Center, Leiden, The Netherlands; Leiden University Center of Infectious Diseases, Leiden University Medical Center, Leiden, The Netherlands; Department of Clinical Pharmacy and Toxicology, Leiden University Medical Center, Leiden, The Netherlands; Division of Systems Pharmacology and Pharmacy, Leiden Academic Centre for Drug Research, Leiden University, Leiden, The Netherlands; Division of Neonatology, Department of Pediatrics, Willem Alexander Children’s Hospital, Leiden University Medical Center, Leiden, The Netherlands

## Abstract

**Background and objectives:**

Early-onset sepsis (EOS) poses a significant morbidity and mortality risk in neonates, for which early diagnosis and adequate antibiotic therapy is crucial. Amoxicillin and benzylpenicillin combined with aminoglycosides are often prescribed empirically for neonatal EOS but optimal dosing regimens are lacking. To evaluate the pharmacokinetics (PK), PTA and toxicity of amoxicillin and benzylpenicillin in (pre)term neonates with EOS, and define optimal dosing regimens.

**Methods:**

One hundred forty-five neonates [gestational age (GA): 24–42 weeks] with EOS treated with intravenous amoxicillin or benzylpenicillin, dosed as per the Dutch Pediatric Formulary (DPF), were included. Amoxicillin and benzylpenicillin were quantified in left-over samples during the first 48 h of life. First, the performance of nine paediatric amoxicillin and benzylpenicillin population PK models was evaluated. Second, the most appropriate models were used for simulation-based PTA and toxicity analyses, evaluating eight international neonatal dosing regimens. Third, simulation-based dose optimization was conducted.

**Results:**

The Bijleveld (amoxicillin) and Padari (benzylpenicillin) models adequately described the obtained PK data (*N* = 252). For amoxicillin, all regimens showed >90% PTA up to 100%fT > MIC but displayed GA-dependent toxicity potential (concentrations >110 mg/L), the DPF regimen excepted. By contrast, all benzylpenicillin regimens showed suboptimal PTA, often accompanied with GA-dependent toxicity potential (concentrations >50 mg/L). Simulations indicated GA-based intermittent dosing or continuous infusion as options to further optimize benzylpenicillin therapy.

**Conclusions:**

(Pre)term neonates with EOS can be adequately treated with amoxicillin dosed as per the DPF regimen. By contrast, further optimization is warranted for benzylpenicillin, for which GA-based intermittent dosing or continuous infusion pose potential alternatives.

## Introduction

Neonatal sepsis remains a predominant cause of morbidity and mortality in newborns.^1^ The incidence of perinatal Group B streptococcus (GBS) infections has risen over recent years in the Netherlands (from 0.20/1000 births in 1987 to 0.32/1000 births in 2011).^1,2^ Timely and adequately dosed antibiotics are thus imperative to minimize adverse outcomes in neonatal sepsis.^3^

However, determination of antimicrobial exposure and efficacy in neonates and particularly preterm neonates is complex as maturational and non-maturational processes yield substantial pharmacokinetic/pharmacodynamic (PK/PD) variability, rendering establishment of appropriate antibiotic dosing regimens challenging. Despite the rise of quantitative PK approaches for antibiotic dose optimization in newborns, comprehensive pharmacometric studies on (pre)term neonates remain scarce, often display limited gestational age (GA) and birth weight distributions and typically lack external validation.^4^ Consequently, current neonatal antibiotic regimens are mainly based on small studies, expert opinion and/or extrapolations from adult studies,^5^ yielding broad and arbitrary dosing recommendations.

Neonatal formularies around the globe display substantial variability in amoxicillin and benzylpenicillin dosing recommendations.^6^ The Dutch Pediatric Formulary (DPF), created to formulate uniform paediatric dosing regimens,^7,8^ proposes a postnatal age (PNA)- (<1 week versus ≥1 week) and birth weight- (<2 kg versus ≥2 kg) based dosing frequency for amoxicillin or benzylpenicillin,^7^ mainly based on a number of small studies.^9–12^ Conversely, NeoFax does not adjust benzylpenicillin dosing for birth weight or PNA (50.000–150.000 IU/kg/day).^13^ By contrast, the Red Book recommends 50.000 IU/kg benzylpenicillin either twice- or thrice-daily, dependent of GA (≤34 versus >34 weeks) and PNA (≤7 versus >7 days).^14^ Clearly, international consensus on the optimal beta-lactam dosing regimens in neonates is lacking.

Therapeutic drug monitoring (TDM)-based individualization of penicillin therapy also comprises a subject of debate, due to the supposed wide therapeutic range of beta-lactam antibiotics.^9^ Given the time-dependent bactericidal effect of beta-lactam antibiotics, the percentage of time that the unbound concentration exceeds the MIC (%fT > MIC) is considered the most informative efficacy parameter. However, there is no scientific consensus which %fT > MIC should be targeted for achieving optimal clinical outcomes. Herein, *in vitro* bactericidal efficacy studies initially substantiated conventional targets of 40%–50%fT > MIC,^15^ but these are gradually shifting towards more rigorous targets of 70%–100%fT > MIC or even 100%fT > 4×MIC.^16–18^ Another complicating factor is that, for most beta-lactam antibiotics, exposure–toxicity relationships are unclear.^19^

We hypothesize that a pharmacometric external evaluation of the current amoxicillin and benzylpenicillin dosing regimens and previously published population PK models for these agents in newborns, in a broad population of extremely preterm to term infants will contribute to further justification and optimization of current dosing regimens.

## Methods

### Patients and samples

The DANCES study included newborns with a GA of 24–42 weeks treated with intravenous gentamicin and amoxicillin or benzylpenicillin for suspected or proven bacteraemia, septicaemia, or meningitis, admitted to the Neonatal Intensive or Medium Care Unit of Leiden University Medical Center (LUMC). Inclusion was enriched for lower GAs by applying GA strata, ceasing recruitment in a stratum when the predefined number of newborns was reached. Sample collection was conducted sparsely from birth to discharge, comprising left-over material from gentamicin TDM or clinical chemistry assessments, conducted as part of routine clinical care. Exclusion criteria included congenital hepatic or kidney disease, infection with a pathogen resistant to amoxicillin and/or benzylpenicillin, or declined written informed consent.

Amoxicillin and benzylpenicillin were dosed as per their respective DPF dosing regimens for severe infection/sepsis.^7^ Accordingly, amoxicillin was given at a 25 mg/kg dose either twice-daily (birth weight <2 kg) or thrice-daily (birth weight ≥2 kg) and benzylpenicillin at 25.000 IU/kg either twice-daily (birth weight <2 kg) or thrice-daily (birth weight ≥2 kg), administered intravenously over 5 min. For each agent, we aimed to collect a total of 120–180 blood samples from 60 patients, with dosing and sampling times registered by the caregiver.

The study was approved by the LUMC Medical Ethical Committee (B20.036) and the parent(s), guardian(s), or legal representative(s) of all participants gave written informed consent.

### Bioanalytics

Amoxicillin and benzylpenicillin were quantified in serum using a previously validated LC-MS/MS assay, used for routine amoxicillin and benzylpenicillin TDM at LUMC.^20^ An overview of its analytical performance is provided in the Supplementary Material (available as Supplementary data at *JAC* Online).

### Population pharmacokinetic analysis

#### General approach

Because of sparse sample collection, development of novel population PK models was considered infeasible. Hence, we (i) identified previously published population PK models for amoxicillin and benzylpenicillin in neonates, (ii) evaluated the ability of these models to describe our PK data, (iii) updated the most appropriate models and (iv) applied these models to perform simulations for dose optimization.

#### Model identification

A PubMed search from inception up to 9 January 2025 was conducted to identify previously published population PK models for amoxicillin and benzylpenicillin in neonates (Supplementary Material), which were translated for application in R package *nlmixr2*.^21,22^

#### Model evaluation

External model evaluation was primarily conducted using goodness-of-fit plots, prediction-corrected visual predictive checks (pcVPCs; *N* = 1000), and normalized prediction distribution errors (NPDEs; *N* = 1000) to test for structural model misspecification. To aid interpretation, predictive performances were also evaluated numerically using (absolute) percentage prediction errors (APPE; PPE), normalized root mean squared errors and the percentages of population-predicted concentrations falling within 10%–30% of the observations (P_10_-P_30_).

#### Model update

If necessary, models were updated through parameter re-estimation based on our PK data, using the FOCEI algorithm in *nlmixr2*. Herein, flow and volume parameters and/or their between-subject variability (BSV) were updated to best fit our population, whereas any covariate relationships from the original models were retained. Updated models were evaluated using the objective function value (OFV), relative standard errors (RSEs), η-shrinkage, goodness-of-fit plots, and simulation-based diagnostics.

#### Simulations

The most appropriate and/or updated population PK models were applied to perform simulations, focusing on the first 48 h of life. Herein, a virtual neonatal population (*N* = 9500) was generated across a GA range of 24–42 weeks including 500 neonates for each whole GA week. For each GA week, birth weights were drawn randomly from GA-matched neonatal birth weight distributions using custom R package *NeoGrowth*,^23,24^ which enables weight simulation from age relying on the Fenton,^25^ Landau-Crangle,^26^ Paul^27^ and Rodd^28^ growth dynamics.

First, the appropriateness of the amoxicillin and benzylpenicillin regimens for neonatal sepsis from eight selected neonatal formularies (Table S1),^29^ including the Australasian Neonatal Medicines Formulary (ANMF),^30^ British National Formulary for Children (BNFC),^31^ DPF,^7^ Pediatric and Neonatal Lexi-Drugs (Lexicomp),^32^ NeoFax,^13^ Neonatal Formulary (NF),^33^ Neonatal Dosage and Practical Guidelines Handbook (NDPGH),^34^ and SwissPedDose (SPD)^35^ were evaluated using PTA analysis. Herein, MIC cut-offs were derived from the EUCAST wildtype cut-off MICs and clinical breakpoints (if applicable) for the typical causative species for early-onset neonatal sepsis and meningitis, including *Streptococcus agalactiae*, *Staphylococcus aureus, Escherichia coli,* and *Listeria monocytogenes* (Table S2).^36–38^ As *S. aureus* displays notorious penicillin antibiotic resistance^39^ and *E. coli* is considered virtually resistant against ampicillin throughout Europe (median 53.5%; range 32.5%–68.6% resistant clones in 2022)^40^ and worldwide (median 71.1%; range 31.7%–100% resistant clones in 2021),^41^ these species are typically covered with aminoglycoside co-therapy (i.e. gentamicin) and were thus omitted from the PTA analysis. The PTA analysis was conducted using unbound drug concentrations, using fixed protein binding constants of 11.7% for amoxicillin^10^ and 49.2% for benzylpenicillin.^42^ As information on the protein binding of beta-lactam antibiotics in neonates is limited, sensitivity analyses were conducted to assess the influence of various degrees of protein binding on the PTA results. Considering the scientific discussion on the optimal PK/PD target for beta-lactam antibiotics, the PTA analysis was conducted over a PK/PD target range of 40%fT > MIC to 100%fT > 4×MIC.^16–18^ A PTA >90% was considered adequate for efficacy.^43,44^

Second, the potential toxicity of the dosing regimens was evaluated. Herein, a toxicity threshold of 110 mg/L was applied for amoxicillin, as amoxicillin concentrations >110 mg/L have been associated with neurotoxicity in adults.^45^ As no clear toxicity thresholds are available for benzylpenicillin, we applied a hypothetical toxicity threshold of >75 mg/L, considering case-reports of benzylpenicillin-associated neurotoxicity at concentrations >75 mg/L in adults.^46,47^ Considering the vulnerability of the developing brain to drug-induced neurotoxicity,^48^ increased unbound fraction during sepsis,^49^ and increased beta-lactam CSF penetration during meningitis,^50^ we also evaluated a > 50 mg/L threshold to screen for prolonged exposure to benzylpenicillin concentrations considered high for adults treated with 12.000.000 IU benzylpenicillin/day.^51^

Third, if deemed necessary based on the PTA and toxicity results, simulation-based dose optimization was conducted.

### Software

Data handling, statistics and graphical visualization were performed in R 4.3.2 (https://www.r-project.org/) and RStudio 2024.12.0 (Posit Software PBC, Boston, MA, USA).

## Results

### Patients and samples

The patient characteristics and PK data are summarized in Table 1. A total of 95 neonates were included for amoxicillin and 50 for benzylpenicillin, with median GAs of 31.9 (range: 24–41) and 31.9 (range: 24–42) weeks, respectively. A total of 170 amoxicillin and 82 benzylpenicillin concentrations were collected.

**Table 1.** Patient and sample information for each drug

	Amoxicillin	Benzylpenicillin
**Patient characteristics**		
Total number of patients, *n*	95	50
Male, *n* (%)	59 (62.11)	29 (58)
Birth classification		
Extremely preterm (GA <28 weeks), *n* (%)	19 (20.00)	11 (22)
Very preterm (GA 28 to <32 weeks), *n* (%)	29 (30.52)	15 (30)
Moderate to late preterm (GA 32 to <37 weeks), *n* (%)	17 (17.89)	9 (18)
Full-term (GA ≥37 weeks), *n* (%)	30 (31.58)	15 (30)
PNA,^a^ median (IQR) [min-max], days	0.11 (0.07–0.30) [0.03–1.96]	0.14 (0.07–0.76) [0.04–5.62]
≤2 days, *n* (%)	95 (100)	45 (90)
>2 and ≤4 days, *n* (%)	0	2 (4)
>4 and ≤6 days, *n* (%)	0	3 (6)
GA, median (IQR) [min-max], weeks	31.86 (29.00–38.00) [24.43–41.29]	31.93 (29.14–38.25) [24.43–41.57]
PMA,^a^ median (IQR) [min-max], weeks	31.99 (29.01–38.11) [24.45–41.30]	31.94 (29.15–38.29) [24.44–42.16]
Birth weight, median (IQR) [min-max], kg	1.83 (1.29–3.14) [0.58–4.91]	1.88 (1.26–3.06) [0.75–4.20]
Admission location		
Neonatal intensive care unit, *n* (%)	85 (89.47)	50 (100)
Neonatal medium care unit, *n* (%)	10 (10.53)	0
Suspected or proven sepsis, *n* (%)^b^	22 (23.16)	16 (32)
Asphyxia,^c^ *n* (%)	16 (16.84)	4 (8)
Apgar score,^d^ median (IQR) [min-max]	8 (7–9) [2–10]^e^	9 (7–9) [4–10]
CRP, median (IQR) [min-max], mg/L	2.00 (0.70–9.65) [0–157]	3.70 (1.65–14.40) [0.20–139.60]
**Pharmacokinetic information**		
Daily dose at treatment initiation		
Absolute dose, median (IQR) [min-max] (mg)	90 (65–240) [30–600]	58 (40–139) [24–297]
mg/kg dose, median (IQR) [min-max] (mg/kg)	51.66 (50–75) [47.3–154.5]	31.86 (30.47–90.67) [28–90.67]
Dosing frequency at treatment initiation		
Twice-daily, *n* (%)	53 (55.79)	28 (56)
Thrice-daily, *n* (%)	42 (44.21)	22 (44)
Total number of samples, *n*	170	82
Number of samples per patient, median (IQR) [min-max]	2 (2–3)[1–4]	2 (1–3)[1–5]

CRP, C-reactive protein; GA, gestational age; IQR, interquartile range; PNA, postnatal age; PMA, postmenstrual age.

^a^Variable measured at start of treatment.

^b^Defined as C-reactive protein >10 mg/L.

^c^Asphyxia, defined as pH <7, Apgar <5, base excess < −16 mmol/L, lactate >10 mmol/L.

^d^Apgar score at 5 min after birth.

^e^Apgar data missing for 2 participants.

### Population pharmacokinetic analysis

#### Model identification

Five relevant neonatal population PK models were identified for amoxicillin^31,52–55^ and four for benzylpenicillin (Tables S3 and S4).^12,31,56,57^ Notably, although only Keij *et al*. and, to a lesser extent, Padari *et al*. comprehensively specified their GA distribution, extremely preterm (GA <28 weeks) and, to a lesser extent, very preterm (GA 28 to <32 weeks) neonates were underrepresented in these studies.

#### Model evaluation and selection

Because of missing covariate information, several assumptions were applied when translating the models to *nlmixr2*. Particularly, current bodyweight was assumed equal to birth weight, body temperature was set to 37°C, and covariate relationships including urine output, serum creatinine or multiorgan failure were normalized.

The Bijleveld model, a one-compartmental model including PMA- and PNA-dependent clearance maturation and allometric scaling to birth weight, best described amoxicillin PK in our population, showing virtually no trends in the goodness-of-fit plots (Figures S1A and S2A), excellent overlap of the simulated and observed data in the pcVPC (Figure S3A) and NPDE (Figure S4A), and superior predictive performance metrics (Table S5). Of note, the Tang model displayed performance similar to the Bijleveld model in smaller neonates (i.e. the twice-daily dosing group), but was discarded because of a misspecification in larger neonates (i.e. the thrice-daily dosing group), and inferior predictive performance metrics. The Barker, Charles, and Keij models were discarded because of notable trends in the goodness-of-fit plots, pcVPC and NPDE misspecifications, and inferior predictive performance.

For benzylpenicillin, the Barker and Muller models exhibited overestimated population predictions compared to the observations. The Bijleveld model showed no systematic bias in the individual predictions and no trends in the goodness-of-fit plots (Figures S1B and S2B), but did not demonstrate adequate overlap between the simulated and observed data in the pcVPC and NPDE (Figures S3B and S4B). Considering the overall performance of the Padari model, including the goodness-of-fit plots, pcVPC and NPDE analyses, and performance metrics (Table S5), this two-compartmental model comprising PMA-dependent clearance maturation and allometric scaling to birth weight, was considered optimal.

#### Model update

For amoxicillin, the Bijleveld model was considered acceptable without modification, despite slight BSV overestimation. As their population and PK dataset were considerably larger than ours, narrowing the BSV through parameter re-estimation was deemed undesirable as this would probably impact generalizability.

For benzylpenicillin, an exploratory update of the Padari model was considered because of apparent BSV underestimation in the pcVPC. However, re-estimation yielded BSV parameters paradoxically lower than those of the original model at relatively high parameter imprecision (Table S6). The Padari model was thus applied without modification.

#### Simulations

**Figure 1.**
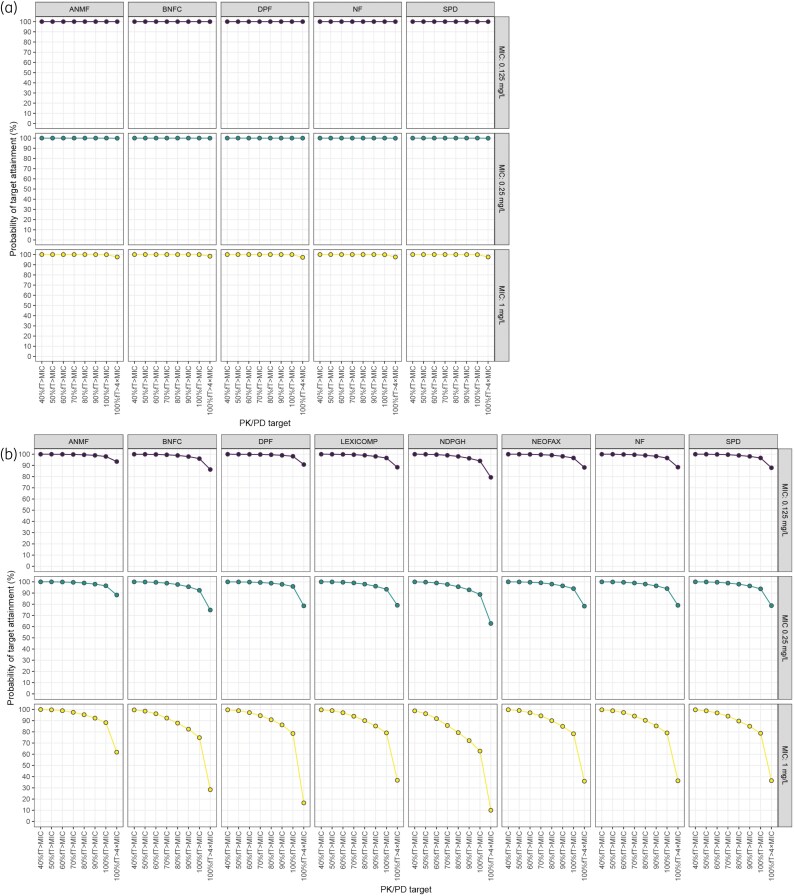
Simulated probability of target attainment (PTA) with the dosing regimens from all current neonatal drug formularies (ANMF, Australasian Neonatal Medicines Formulary; BNFC, British National Formulary for Children; DPF, Dutch/German/Austrian/Norwegian Pediatric Formularies; Lexicomp, Pediatric and Neonatal Lexi-Drugs; NeoFax; NF, Neonatal Formulary; NDPDH, Neonatal Dosage and Practical Guidelines Handbook; SPD, SwissPedDose) for each MIC and %fT > MIC threshold for a) amoxicillin and b) benzylpenicillin.

**Figure 2.**
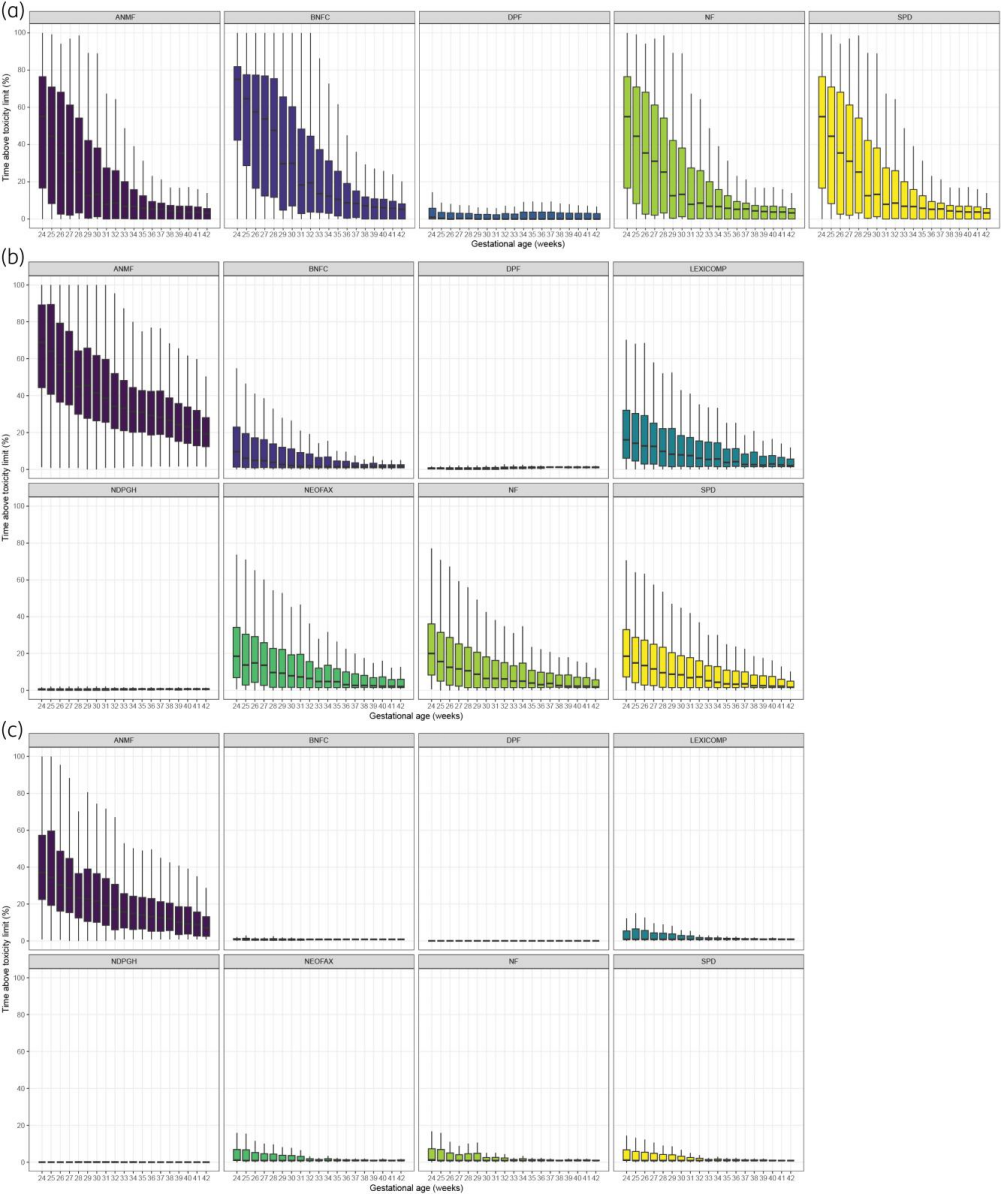
Simulated percentage of time during the first 48 h of life that neonates display concentrations above the toxicity limit for all current neonatal drug formulary dosing regimens (ANMF, Australasian Neonatal Medicines Formulary; BNFC, British National Formulary for Children; DNF, Dutch/German/Austrian/Norwegian Pediatric Formularies; Lexicomp, Pediatric and Neonatal Lexi-Drugs; NeoFax; NF, Neonatal Formulary; NDPDH, Neonatal Dosage and Practical Guidelines Handbook; SPD, SwissPedDose), for each gestational age (GA) category for a) amoxicillin, b) benzylpenicillin with a toxicity threshold of 50 mg/L and c) benzylpenicillin with a toxicity threshold of 75 mg/L.

No comprehensive dosing regimens for intravenous amoxicillin were available in the Lexicomp, NeoFax, and NDPGH formularies. For the available formularies, the PTA analysis showed excellent target attainment across all MICs, with PTAs of 100% for the 40%–100%fT > MIC range and >90% for 100%fT > 4×MIC (Figure 1a, Table S7). Protein binding perturbation (5%–15%) yielded similar PTA results (Table S8). These PTA results were often accompanied with prolonged exposure to amoxicillin concentrations >110 mg/L, particularly for the ANMF, BNFC, NF and SPD regimens (Figure 2a). These regimens displayed distinct trends between GA and overexposure, with the ANMF, BNFC, NF and SPD regimens showing respective median durations of overexposure of 3.3%–8.5%, 5.3%–19.4%, 3.3%–8.5% and 3.3%–8.5% of the first 48 h of life in neonates with a GA ≥32 weeks, but rendering extremely and very preterm neonates (GA <32 weeks) exposed to potentially toxic amoxicillin concentrations for median durations of 7.9%–55.0%, 18.2%–75.1%, 7.9%–55.0% and 7.9%–55.0%, respectively. Contrarily, the DPF regimen showed limited toxicity potential, yielding median overexposures between 0.4% and 1.0% across the entire GA range.

For benzylpenicillin, all formularies were evaluated. The PTA analysis demonstrated excellent target attainment for the 0.125 mg/L and 0.25 mg/L MIC breakpoints, with PTA >90% for the 40%–100%fT > MIC range across all formularies. For the 100%fT > 4×MIC target, >90% PTA was only reached for the 0.125 mg/L MIC breakpoint with the ANMF regimen (Figure 1b, Table S7). PTA for the 1 mg/L MIC breakpoint was variable, with none of the formularies reaching >90% PTA up to 100%fT > MIC. The AMNF regimen reached >90% PTA up to 90%fT > MIC, the BNFC, DPF, Lexicomp, NeoFax, NF and SPD regimens up to 80%fT > MIC, and the NDPGH regimen up to 60%fT > MIC (Figure 1b). Protein binding perturbation (40%–60%) yielded similar PTA results (<±5% deviation) for most PK/PD targets and MIC breakpoints (Table S8). However, it revealed more uncertainty at low PTA, especially with the 100%fT > MIC and 100%fT > 4xMIC targets at the 1 mg/L MIC breakpoint (Table S8). Regarding potential overexposure, all formularies except the DPF and NDPGH showed distinct trends of increasing exposure to benzylpenicillin concentrations >50 mg/L with decreasing GA (Figures 2b). Exposure to concentrations >75 mg/L showed a similar trend over GA for the ANMF regimen, but was limited for the other formularies (Figures 2c).

**Figure 3.**
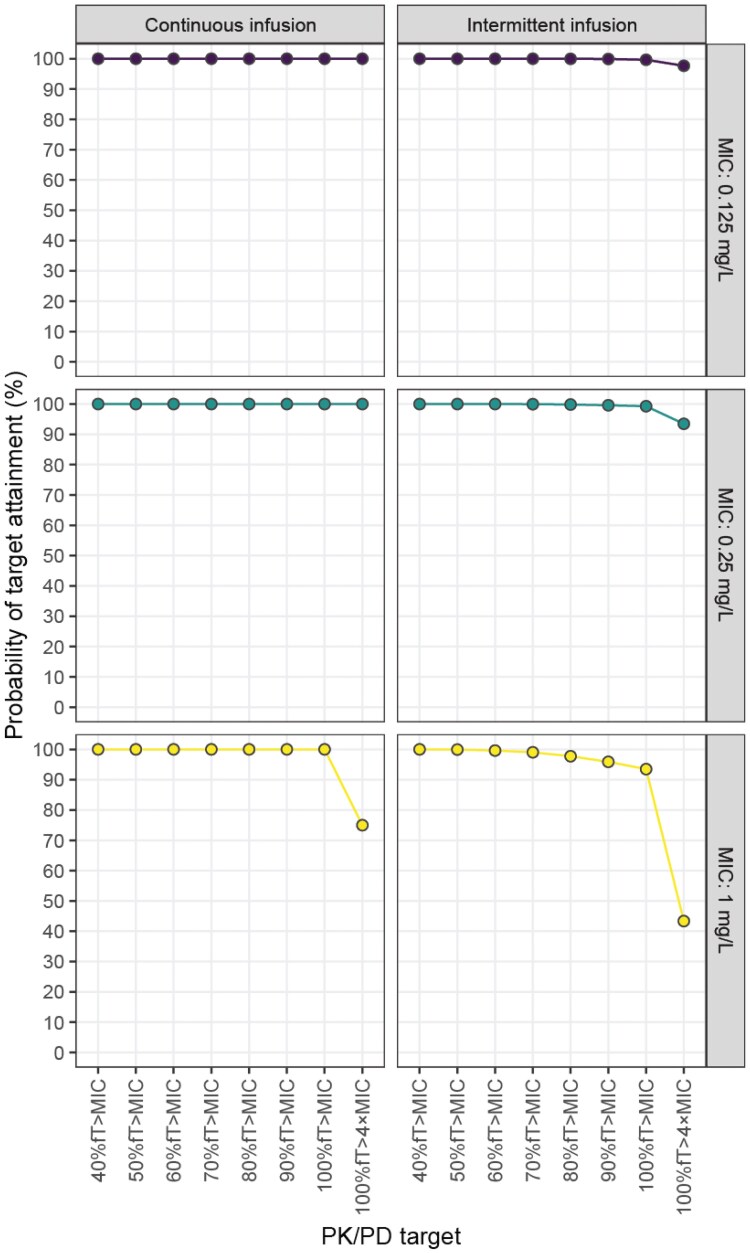
Simulated probability of target attainment (PTA) with the alternative intermittent infusion and continuous infusion dosing regimens for benzylpenicillin, for each MIC and %fT > MIC threshold, assuming 49.2% protein binding.

**Figure 4.**
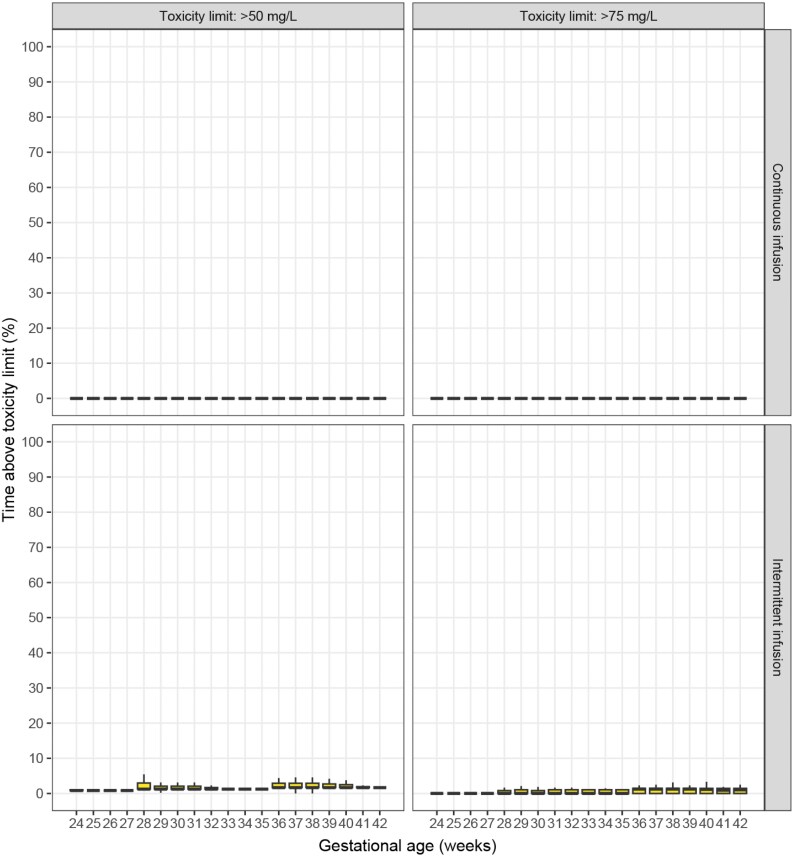
Simulated percentage of time during the first 48 h of life that neonates display concentrations above the 50 mg/L and 75 mg/L toxicity limits with the alternative intermittent infusion and continuous infusion dosing regimens for benzylpenicillin, for each gestational age (GA) category.

As all benzylpenicillin regimens showed suboptimal PTA often accompanied with potential overexposure, alternative dosing regimens were explored using the DPF regimen as a starting point. Explorative simulations with the DPF regimen indicated inadequate GA adjustment, displaying GA-dependent trends in underexposure (Figure S5A). Additionally, the simulated maximal concentrations indicated room for a modest dose increase (Figure S5B). Indeed, a regimen comprising a GA-dependent dosing frequency with a 30.000 IU/kg dose every 12 h (GA <28 weeks), 8 h (GA ≥28 to <36 weeks) or 6 h (GA >36 weeks), showed >90% PTA up to 100%fT > MIC for all MIC breakpoints (Figures 3a and S6A, Table S9) with limited potential toxicity (Figures 4 and S6B). As previously suggested,^58^ continuous infusion with a loading dose was also explored. Herein, continuous infusion of the DPF-based total daily dose, combined with a loading dose of 16.7% of the total daily dose, yielded 100% PTA up to 100%fT > MIC for all MIC breakpoints (Figure 3 and S6C, Table S9), with virtually no potential toxicity (Figure 4 and S6D). Sensitivity analyses with 40%–60% protein binding yielded similar results (Table S10).

## Discussion

We externally evaluated nine previously published amoxicillin and benzylpenicillin population PK models in extremely preterm to full-term neonates, showing variable ability to describe their PK across the neonatal population. The Bijleveld^53^ and Padari^57^ models showed optimal predictive performance for amoxicillin and benzylpenicillin, respectively, despite showing slight BSV over- and underestimation. For amoxicillin (if combined with gentamicin to treat pathogens not sensitive to amoxicillin), all regimens showed excellent coverage of the typical causative species for neonatal sepsis, even when considering PK/PD targets up to 100%fT > 4×MIC. However, this was often accompanied with extensive exposure to potentially toxic amoxicillin concentrations in prematurely born neonates, with most dosing regimens showing trends of increasing amoxicillin overexposure with decreasing GA. Overall, the DPF regimen showed distinct superiority over the other formularies, yielding excellent PTA with limited toxicity potential. Clearly, a lower dose (i.e. 25 mg/kg) combined with a weight-based dosing frequency (i.e. <2 kg, twice-daily;  ≥ 2 kg, thrice-daily), comprises a superior dosing strategy for the first 48 h of life as compared to higher doses combined with a uniform dosing frequency irrespective of birth weight (i.e. 50–60 mg/kg twice-daily). For benzylpenicillin (if combined with gentamicin to treat pathogens not sensitive to benzylpenicillin), the current neonatal dosing regimens displayed adequate PTA up to 100%fT > MIC for *S. agalactiae*, but lower PTA (>90% PTA up to 60%–80%fT > MIC) for *L. monocytogenes*. Furthermore, all current regimens except the DPF and NDPGH showed distinct trends of increasing benzylpenicillin overexposure with decreasing GA, rendering extremely preterm and, to a lesser extent, very preterm neonates exposed to extensive benzylpenicillin overexposure.

Whereas the DPF dosing regimen seems adequate for amoxicillin, our findings warranted further optimization of benzylpenicillin therapy for early-onset neonatal sepsis, particularly when aiming to cover *L. monocytogenes* although this pathogen may be considered not a target for penicillin. Whereas a 40%–50%fT > MIC PK/PD target may be acceptable for non-severe infections in less fragile patient populations, higher PK/PD targets seem desirable for severe infections in neonates.^58,59^ We showed that intermittent infusion at a GA-based frequency or continuous infusion with a loading dose pose promising alternatives to improve PTA with limited toxicity potential.

Previous studies on this topic have yielded variable results. Leegwater *et al*.^58^ showed inadequate 100%fT > MIC attainment with the DPF regimen for the first 48 h of amoxicillin and benzylpenicillin treatment in neonates aged 0–14 days, using MIC breakpoints of 8 mg/L and 2 mg/L for amoxicillin and benzylpenicillin, respectively. These authors proposed continuous infusion with a loading dose to improve target attainment for amoxicillin and benzylpenicillin. For the antibiotic treatment of (suspected) neonatal sepsis in neonates aged 0–2 days, our findings suggest that continuous infusion with a loading dose indeed seems promising for benzylpenicillin but does not seem required for amoxicillin. Gastine *et al*.^59^ evaluated the PTA and toxicity of several beta-lactam antibiotics including amoxicillin and benzylpenicillin, using various international paediatric dosing formularies. Simulations were conducted for a virtual paediatric population aged 23 weeks PMA to 18 years PNA with PTA evaluated over the first 24 h after reaching steady-state, using 40%fT > MIC and 100%fT > MIC as PK/PD targets over the entire MIC range for susceptible and resistant pathogens causing sepsis, pneumonia, and meningitis.^59^ Although the differences with our PTA definitions render direct comparison difficult, their results also indicated adequate median PTA for amoxicillin at MICs of 0.125–1 mg/L in neonatal sepsis.^59^ Benzylpenicillin showed inadequate PTA for MICs >0.125 mg/L for neonatal pneumonia.^59^ Van Donge *et al*.^60^ evaluated amoxicillin toxicity and PTA for MICs of 0.25–16 mg/L and PK/PD targets of 30%–100%fT > MIC for the first 7 days of treatment in neonates aged 0–60 days PNA, using the Tang model.^54^ Although we demonstrated that the DPF regimen yields adequate amoxicillin PTA with limited toxicity potential for (suspected) neonatal sepsis across the entire GA range during the first 48 h of life, these authors reported lower PTA over the first 7 days following treatment initiation in neonates and young infants aged 0–60 days.^60^

This study has several limitations. First, although we demonstrated that the Bijleveld and Padari models adequately describe amoxicillin and benzylpenicillin PK in (pre)term neonates, they displayed slight BSV over- and underestimation. Hence, the adequacy of the proposed dosing regimens should be confirmed clinically before widespread application. Second, our toxicity analyses were based on threshold concentrations from adult literature. Whereas the adult literature already displays high heterogeneity regarding beta-lactam neurotoxicity, extrapolation to neonates is complex. However, as the developing brain is considered more vulnerable to drug-induced neurotoxicity,^48^ it seems likely that beta-lactam concentrations associated with neurotoxicity in adults will also evoke adverse neurological effects in neonates. Moreover, meningitis often complicating neonatal sepsis may render critically ill neonates particularly susceptible to beta-lactam-associated neurotoxicity due to increased CSF penetration during meningeal inflammation,^50^ and sepsis-associated hypoalbuminemia may result in higher unbound concentrations,^49^ in a population already displaying lower protein binding than observed in adults.^61^ Also, beta-lactam-associated neurotoxicity is likely underreported in neonates due to complex seizure detection in this population.^50^ Altogether, this warrants a cautious approach and minimization of beta-lactam overexposure in neonates. Third, amoxicillin and benzylpenicillin were quantified as total concentrations, with the PTA analysis conducted using fixed protein binding constants derived from two small studies.^10,42^ Although debatable, these protein binding percentages do align with alternative approaches relying on prediction of neonatal protein binding from adult values.^61^ Notably, fixed protein binding constants inherently ignore the actual neonatal protein binding distribution, typically resulting in underestimated target attainment.^62^ Nevertheless, our sensitivity analyses showed a limited effect of protein binding perturbation on PTA. Fourth, divergences between the performance of our bioanalytical assay and those of the evaluated studies may have introduced systematic biases in our external evaluation.^63,64^ However, as the maximal divergences between the reported assay biases and those of our assay were approximately ±18% for amoxicillin^31,52,53,55^ and ±15% benzylpenicillin,^31,56,57^ inter-assay heterogeneity likely did not substantially affect our analyses. Finally, it is important to acknowledge that our neonatal population reflects one from a high income country, which may limit the generalizability of our results to populations displaying similar incidences of actual neonatal sepsis and/or meningitis and similar antimicrobial resistance patterns.

### Conclusion

We externally evaluated nine previously published population PK models for amoxicillin and benzylpenicillin in neonates, and assessed the adequacy of eight international neonatal dosing guidelines for these drugs for (suspected) early-onset neonatal sepsis. For amoxicillin, the DPF regimen showed adequate efficacy and safety during the first 48 h of life, across the entire population of extremely preterm to full-term neonates. For benzylpenicillin, all regimens showed suboptimal efficacy, often accompanied with GA-dependent overexposure. Using simulations, we showed that GA-dependent dosing regimens provide promising alternatives for further clinical exploration.

## Supplementary Material

dkaf191_Supplementary_Data

## Data Availability

All data from this study are available from the corresponding author upon reasonable request. The *nlmixr2* model code of all applied population PK models is available in the Supplementary Material.

## Supplementary data

Figures S1 to S5 and Tables S1 to S10 are available as Supplementary data at *JAC* Online.
