# Supplementary material for: Evaluation of amoxicillin and benzylpenicillin therapy in early-onset neonatal sepsis: a pharmacometric external validation and simulation study
Source: J Antimicrob Chemother. 2025 Jun 16;80(8):2214–25. doi: 10.1093/jac/dkaf191 (PMC12313450; doi:10.1093/jac/dkaf191)
Supplement: dkaf191_Supplementary_Data [file dkaf191_supplementary_data.docx]

Supplementary material

**Evaluation of amoxicillin and benzylpenicillin therapy in early-onset neonatal sepsis: a pharmacometric external validation and simulation study**

**Authors**

Tom C Zwart^1,2*^, Dimitra Eleftheriou^3*^, Sophie J Jansen^4^, Martha T van der Beek^5^, Dirk Jan AR Moes^1^, Swantje Völler^3✝^, Vincent Bekker^4✝^

*These authors contributed equally

✝These authors contributed equally

**Author affiliations**

1. Department of Clinical Pharmacy & Toxicology, Leiden University Medical Center, Leiden, The Netherlands.
2. Department of Clinical Pharmacy, Haga Teaching Hospital, The Hague, The Netherlands.
3. Division of Systems Pharmacology and Pharmacy, Leiden Academic Centre for Drug Research, Leiden University, Leiden, The Netherlands.
4. Division of Neonatology, Department of Pediatrics, Willem Alexander Children’s Hospital, Leiden University Medical Center, Leiden, The Netherlands.
5. Department of Medical Microbiology, Leiden University Medical Center, Leiden, The Netherlands

**Supplementary figures**

**Figure S1.** Goodness-of-fit plots for all evaluated models for a) amoxicillin, and b) benzylpenicillin. Black solid lines and grey-shaded areas represent loess regression fits and their standard errors, respectively.

**
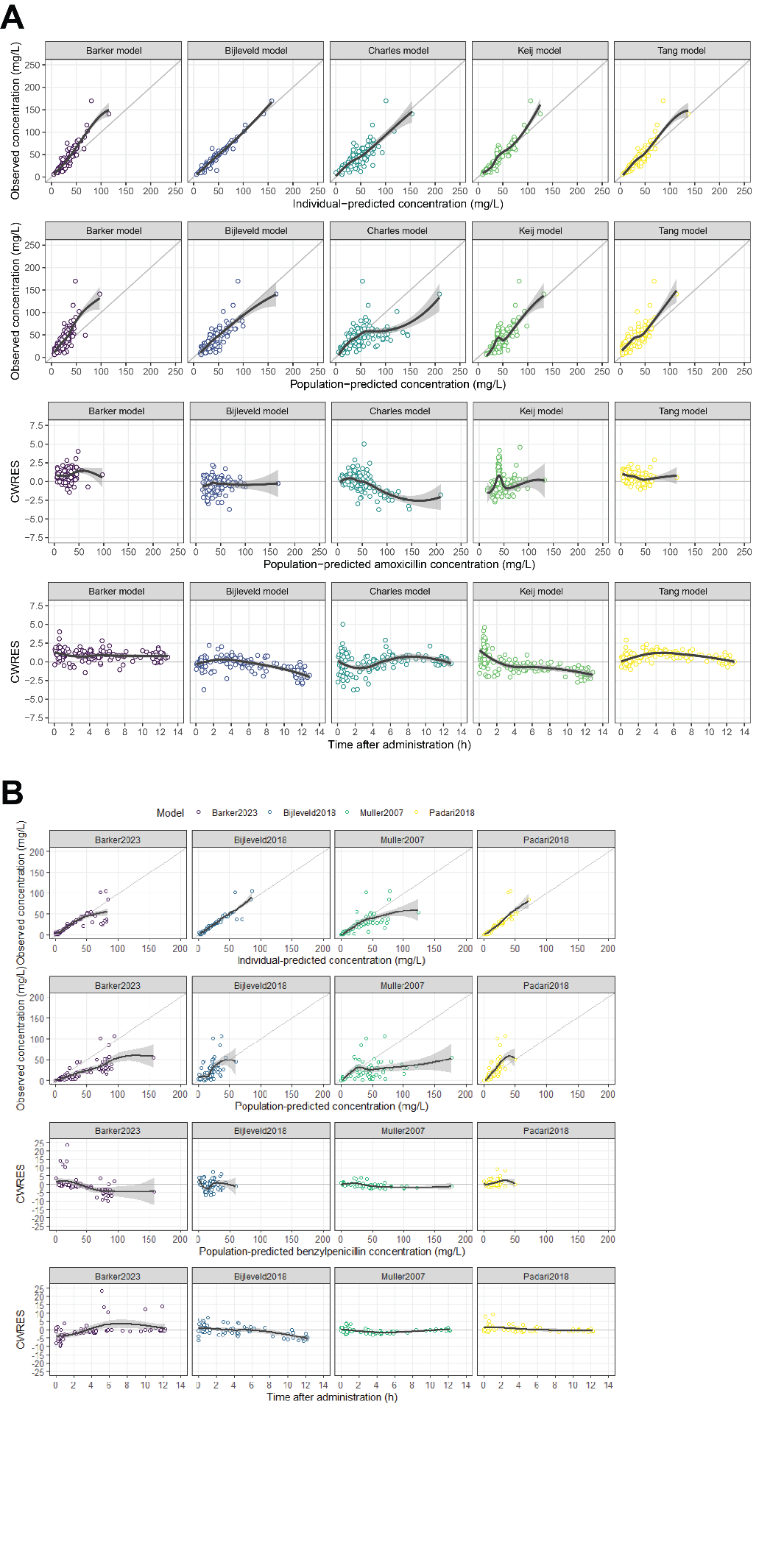
**

**Figure S2.** Conditional-weighted residuals (CWRES) over gestational age and birth weight for all evaluated models for a) amoxicillin, and b) benzylpenicillin. Black solid lines and grey-shaded areas represent loess regression fits and their standard errors, respectively.

**
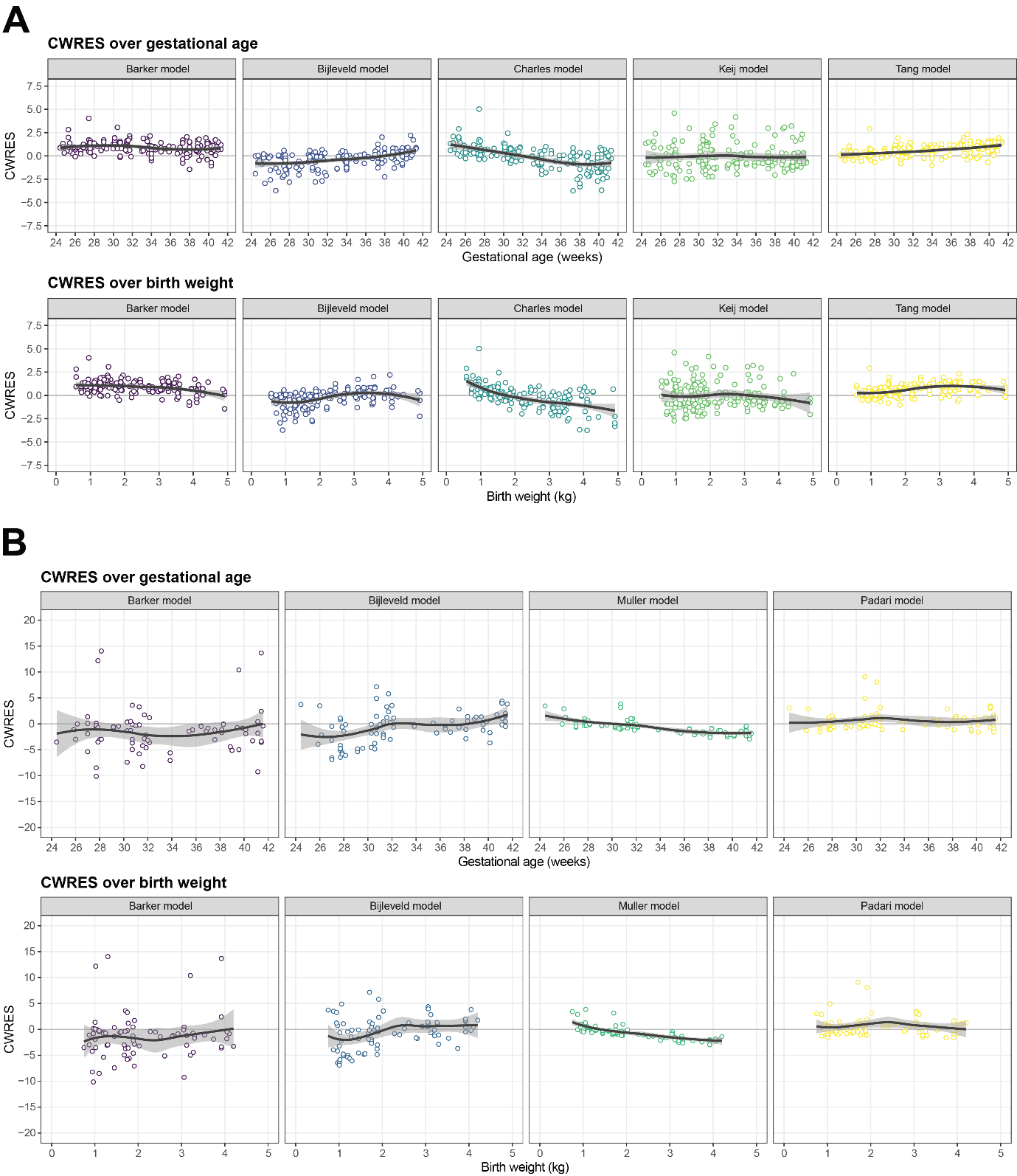
**

**Figure S3.** Prediction-corrected visual predictive checks (pcVPCs) for all evaluated models, stratified according to the dosing frequency applied in the clinical study, for a) amoxicillin, and b) benzylpenicillin. Solid and dashed black lines represent the median, 5th and 95th percentiles of the observed PK data. Dark and light blue-shaded areas represent the 95% confidence intervals around the median, 5th and 95th percentiles of the simulated PK data.

**
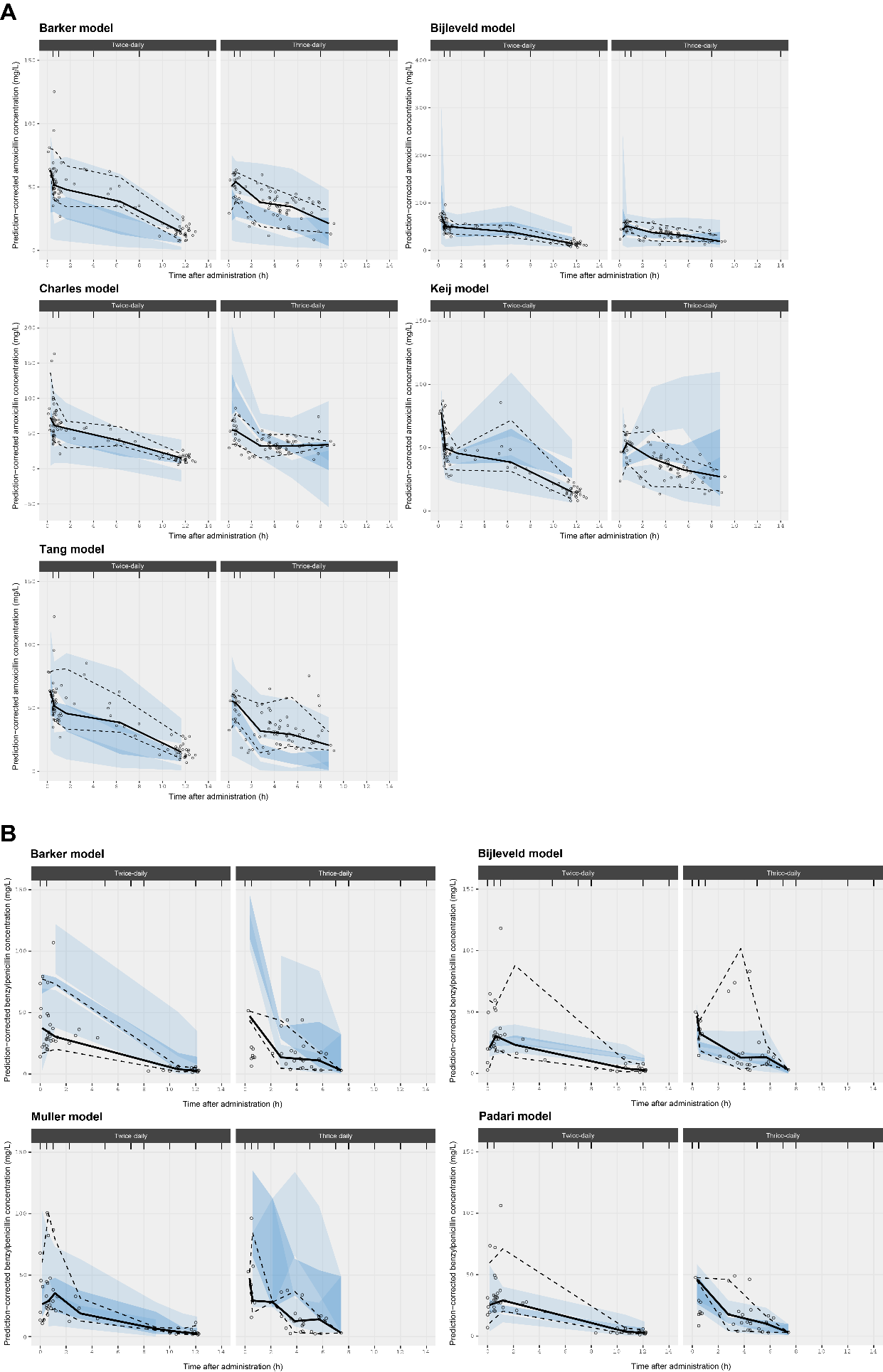
**

**Figure S4.** Normalized prediction distribution error (NPDE) plots all evaluated models for a) amoxicillin, and b) benzylpenicillin.

**
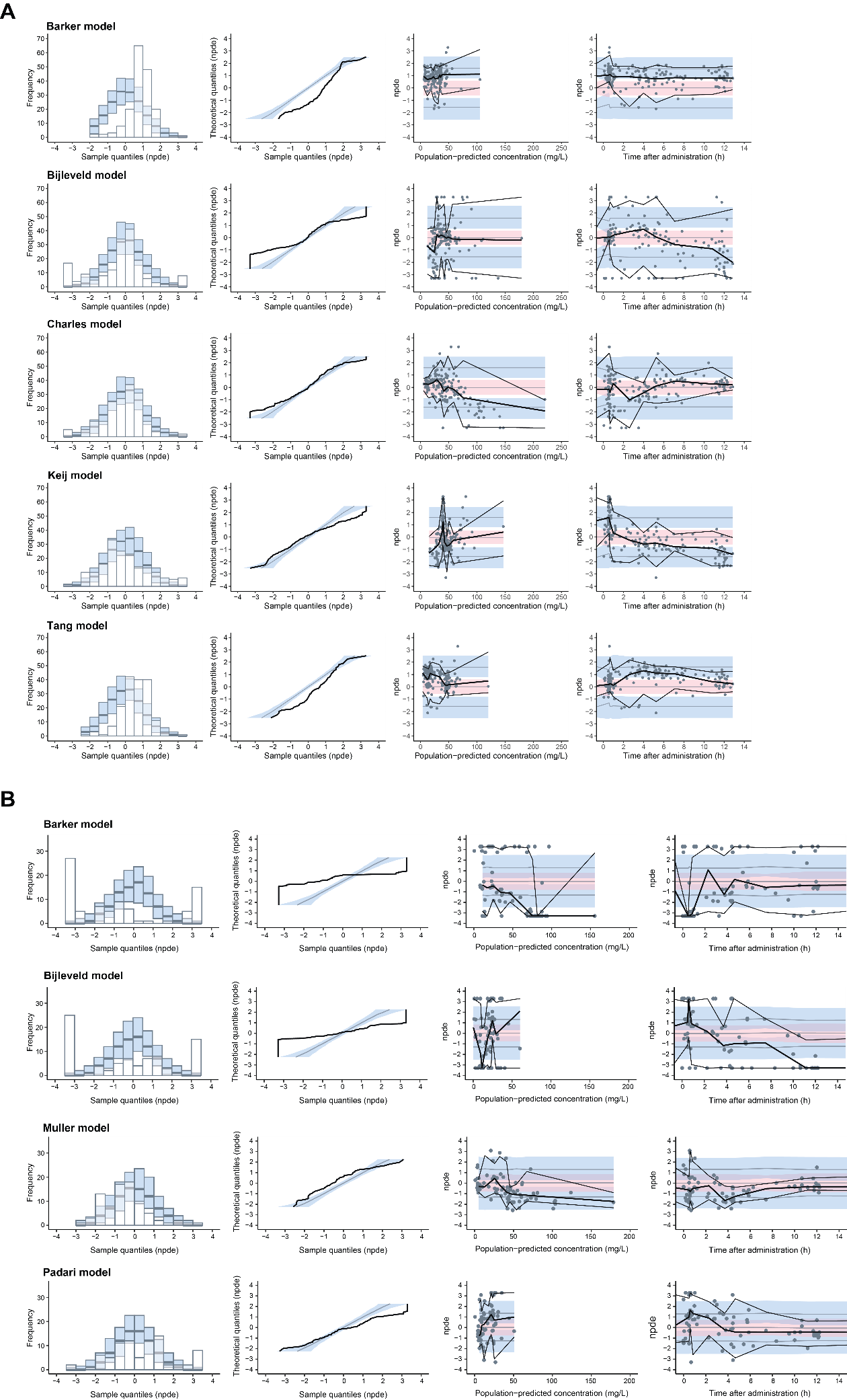
**

**Figure S5.** Simulated a) unbound and b) total benzylpenicillin concentration-time profiles for the first 48 h of life with the current Dutch Pediatric Formulary (DPF) regimen, stratified per birth weight (<2 kg versus ≥2 kg) category and gestational age (GA) category, using 49.2% protein binding and a MIC breakpoint of 1 mg/L for efficacy and a cut-off of 75 mg/L for toxicity. For each GA stratum, the blue solid line and blue-shaded area depict the median predicted benzylpenicillin concentration and its 90% prediction interval, respectively.


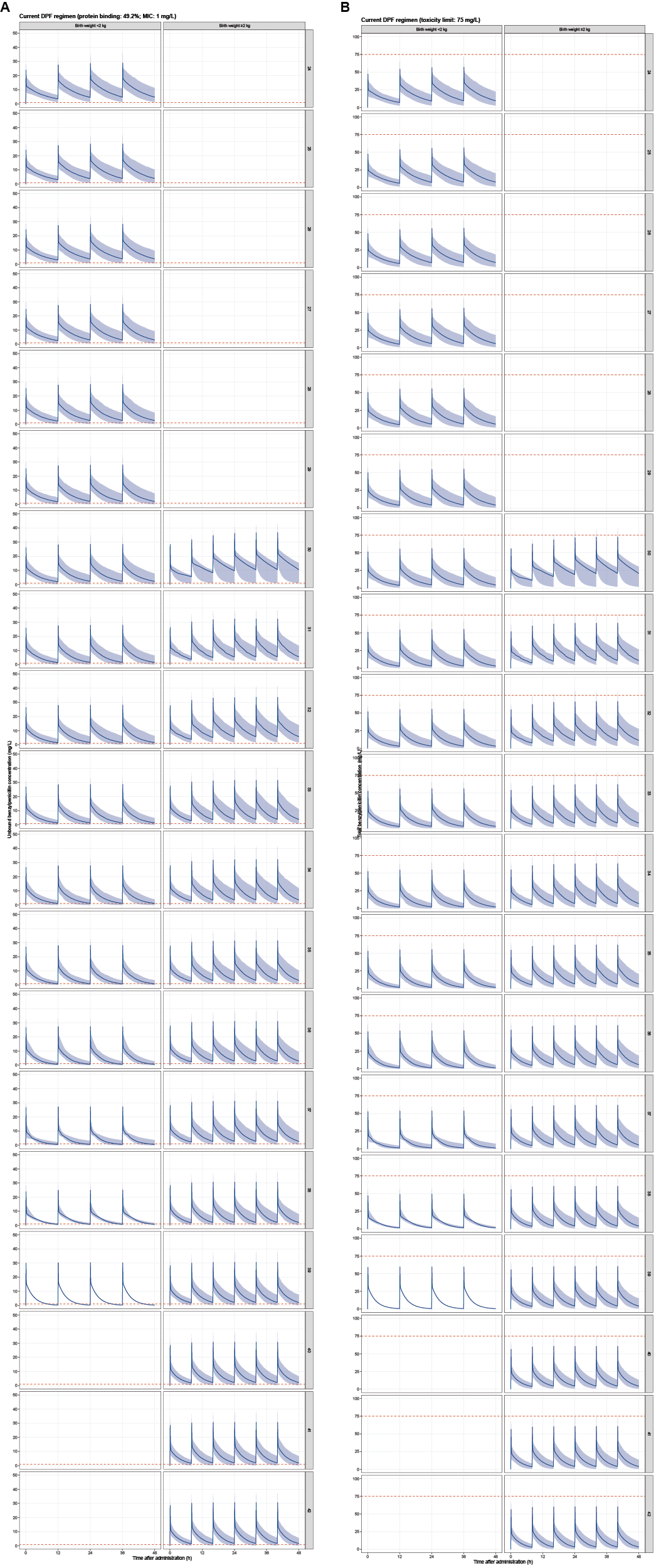
­

**Figure S6.** Simulated a) unbound and b) total benzylpenicillin concentration-time profiles with the alternative intermittent infusion regimen, and simulated c) unbound and d) total benzylpenicillin concentration-time profiles with the alternative continuous infusion regimen for the first 48 h of life, stratified per gestational age (GA) category, using 49.2% protein binding and a MIC breakpoint of 1 mg/L for efficacy and a cut-off of 75 mg/L for toxicity. For each GA stratum, the blue solid line and blue-shaded area depict the median predicted benzylpenicillin concentration and its 90% prediction interval, respectively.


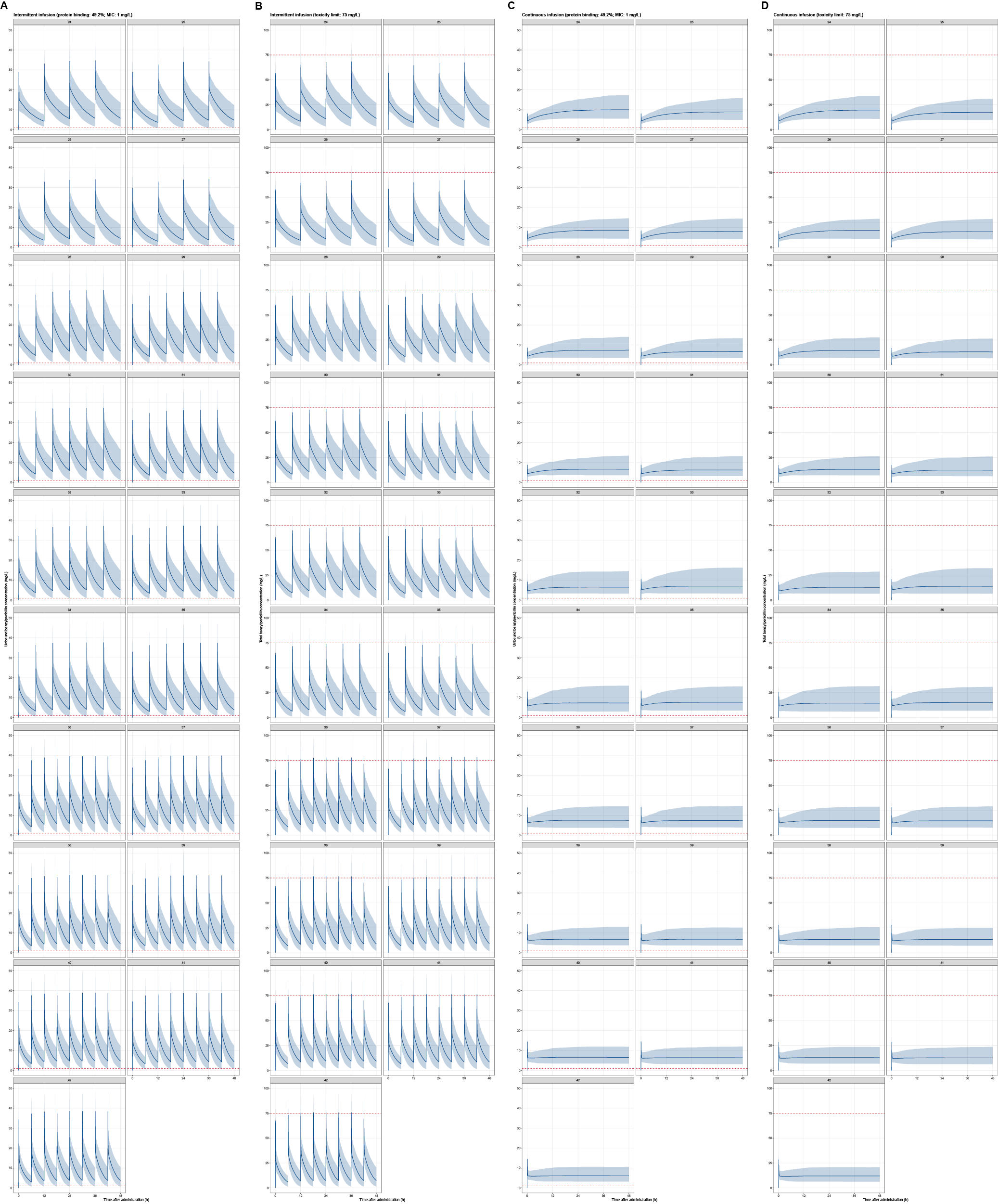


**­­­**

**Supplementary tables**

**Table S1.** Overview of neonatal drug formularies and concurrent dosing regimens for intravenous amoxicillin and benzylpenicillin therapy for (suspected) neonatal sepsis.

| **Formulary** | **Amoxicillin** | **Benzylpenicillin** |
| --- | --- | --- |
| Australasian Neonatal Medicines Formulary  (ANMF)^1^ | GA <30 weeks; PNA ≤28 days: 50 mg/kg q12h  GA <30 weeks; PNA >28 days: 50 mg/kg q8h  GA ≥30-≤36 weeks; PNA ≤14 days: 50 mg/kg q12h  GA ≥30-≤36 weeks; PNA >14 days: 50 mg/kg q8h  GA >37 weeks; PNA ≤7 days: 50 mg/kg q12h  GA >37 weeks; PNA >7 days: 50 mg/kg q8h | GA <30 weeks; PNA ≤28 days: 60 mg/kg q12h  GA <30 weeks; PNA >28 days: 60 mg/kg q8h  GA ≥30-≤36 weeks; PNA ≤14 days: 60 mg/kg q12h  GA ≥30-≤36 weeks; PNA >14 days: 60 mg/kg q8h  GA >37 weeks; PNA ≤7 days: 60 mg/kg q12h  GA >37 weeks; PNA >7 days: 60 mg/kg q8h |
| British National Formulary for Children  (BNFC) 2022^2^ | PNA <7 days: 30-60 mg/kg q12h^a^  PNA 7-≤28 days: 30-60 mg/kg q8h^a^  PNA >28 days: 20-60 mg/kg (max. 500 mg) q8h^a^ | PNA <7 days: 25 mg/kg q8h-q12h^b^  PNA 7-≤28 days: 25-50 mg/kg q8h^b^  PNA >28 days: 25-50 mg/kg (max. 2400 mg) q4h-q6h^b^ |
| Dutch/German/Austrian/Norwegian Pediatric Formularies (DPF)^3^ | BW <2 kg;PNA <7 days: 25 mg/kg q12h  BW ≥2 kg; PNA <7 days: 25 mg/kg q8h  BW <2 kg; PNA ≥7 to <28 days: 25 mg/kg q8h  BW ≥2 kg; PNA ≥7 to <28 days: 25 mg/kg q12h  PNA ≥28 days: 25 mg/kg q6h or 33.3 mg/kg q8h^c^ | BW <2 kg; PNA <7 days: 25.000 IU/kg q12h  BW ≥2 kg; PNA <7 days: 25.000 IU/kg q8h  BW <2 kg; PNA ≥7 to <28 days: 25.000 IU/kg q8h  BW ≥2 kg; PNA ≥7 to <28 days: 25.000 IU/kg q6h  PNA ≥28 days: 25.000-100.000 IU/kg q6h or 16.666-66.666 IU/kg q4h^d^ |
| Pediatric and Neonatal Lexi-Drugs (Lexicomp)^4^ | BW <1.5 kg: NR  BW 1.5 to <2 kg: 37.5 mg/kg q12h  BW ≥2 kg: 50 mg/kg q12h | GA ≤34 weeks; PNA ≤7 days: 50.000 IU/kg q12h  GA ≤34 weeks; PNA >7 days: 50.000 IU/kg q8h  GA >34 weeks; PNA ≤7 days: 50.000 IU/kg q12h  GA >34 weeks; PNA >7 days: 50.000 IU/kg q8h |
| NeoFax^5^ | NR^e^ | PMA ≤29 weeks; PNA 0-≤28 days: 25.000-50.000 IU/kg q12h^f^  PMA ≤29 weeks; PNA >28 days: 25.000-50.000 IU/kg q8h^f^  PMA 30-≤36 weeks; PNA 0-≤14 days: 25.000-50.000 IU/kg q12h^f^  PMA 30-≤36 weeks; PNA >14 days: 25.000-50.000 IU/kg q8h^f^  PMA 37-≤44 weeks; PNA 0-≤7 days: 25.000-50.000 IU/kg q12h^f^  PMA 37-≤44 weeks; PNA >7 days: 25.000-50.000 IU/kg q8h^f^  PMA >45 weeks: 25.000-50.000 IU/kg q6h^f^ |
| Neonatal Formulary (NF)^6^ | PNA 0-<7 days: 50 mg/kg q12h  PNA ≥7-<28 days: 50 mg/kg q8h  PNA ≥28 days: 50 mg/kg q6h | PNA 0-<7 days: 30 mg/kg q12h  PNA ≤7-<28 days: 30 mg/kg q8h  PNA ≥28 days: 30 mg/kg q6h |
| Neonatal Dosage and Practical Guidelines Handbook (NDPGH)^7^ | NR^c^ | PNA ≤7 days; BW ≤2 kg: 25.000 IU/kg q12h  PNA ≤7 days; BW >2 kg: 25.000 IU/kg q8h  PNA >7 days; BW <1.2 kg: 25.000 IU/kg q12h  PNA >7 days; BW ≥1.2 to <2 kg: 25.000 IU/kg q8h  PNA >7 days; BW ≥2 kg: 25.000 IU/kg q6h |
| SwissPedDose (SPD)^8^ | PNA <7 days: 50 mg/kg q12h  PNA ≥7 to <28 days: 50 mg/kg q8h | PNA <7 days: 50.000 IU/kg q12h  PNA ≥7 to <28 days: 50.000 IU/kg q8h |

BW, weight at birth; GA, gestational age; NR, not reported; PMA, postmenstrual age; PNA, postnatal age.

^a^ Simulations were conducted with the high-dose regimen for severe infection.

^b^ Simulations were conducted with the high-dose regimen and shortest dosing interval for severe infection.

^c^ Up to a maximum of 12g/day.

^d^ Up to a maximum of 24.000.000 IU/day.

^e^ Only oral dosing information available for amoxicillin.

^f^ Simulations were conducted with the high-dose regimen for severe infection.

**Table S2.** Selected MIC breakpoints, as derived from the EUCAST database.^9^

| **Species** | **Drug** | **Wildtype MIC**  (mg/L) | **Indication** | **Clinical breakpoint**  (mg/L) |
| --- | --- | --- | --- | --- |
| *S. agalactiae* | Amoxicillin | ≤0.125 | Meningitis | Inferred from benzylpenicillin |
|  | Ampicillin | ≤0.25 | Meningitis | Inferred from benzylpenicillin |
|  | Benzylpenicillin | ≤0.125 | Sepsis | ≤0.25 |
|  |  |  | Meningitis | ≤0.125 |
| *L. monocytogenes* | Amoxicillin | - | Meningitis | - |
|  | Ampicillin | ≤1 | Meningitis | ≤1 |
|  | Benzylpenicillin | ≤1 | Sepsis | ≤1 |
|  |  |  | Meningitis | Insufficient evidence |

**Table S3**. Overview of patient characteristics and pharmacokinetic data of all evaluated population pharmacokinetic studies.

| **Drug** | **Study** | **N** | **GA**  (weeks) | **PNA**  (days) | **Birthweight**  (kg) | **Dosing regimen** | **Pharmacokinetic data** |
| --- | --- | --- | --- | --- | --- | --- | --- |
| Amoxicillin | Barker *et al*. 2023^2^ | 174 | 38.6 (22.86–41.86) | 50 (0.7–5711) | 3.8 (0.6-70)^a^ | *Susceptible infections*  PNA <7 days: 30 mg/kg q12h  PNA 7-28 days: 30 mg/kg q8h  PNA >1 month: 20–60 mg/kg q8h | N=409, 0-24 h after administration |
|  |  |  |  |  |  | *Listeria meningitis*  PNA <7 days: 50-100 mg/kg q12h  PNA 7-28 days: 50-100 mg/kg q8h  PNA >1 month: 50-100 mg/kg q6h or q4h |  |
|  | Bijleveld *et al*. 2018^10^ | 125 | 40 (36-42) | 5 (2-5) | 3.3 (2.1-5.1) | Majority received either 50 mg/kg q12h (61.6%) or 25 mg/kg q8h (32.8%) | N=1280, 0-70 h after administration |
|  | Charles *et al*. 1997^11^ | 40 | 28.9 (24-32) | 1.1 (1-3) | 1.1 (0.6-1.5) | 50 mg/kg q12h | N=214, 0-50 h after administration |
|  | Keij *et al*. 2023^12^ | 261 | 37.4 (IQR 31.7–39.86) | 1 (IQR 0–4) | 2.6 (IQR 1.6–3.5)^a^ | Median 50 mg/kg (range: 9.4–112.9) | N=938, 0-16 h after administration |
|  | Tang *et al*. 2019^13^ | 187 | 38 (28-41) | 7 (1-37) | 3.1 (1.0-4.6) | 25 mg/kg q12h | N=224, 0-16 h after administration |
| Benzylpenicillin | Barker *et al*. 2023^2^ | 64 | 37 (24-42.29) | 2.1 (0.7–4795.7) | 2.83 (0.57-64.3)^b^ | *Sepsis*  PNA < 7 days: 25 mg/kg q8h or q12h  PNA 7-28 days: 25-50 mg/kg q8h  PNA > 28 days: 25-50 mg/kg q4h or q6h | N=147, 0-24 h after administration |
|  |  |  |  |  |  | *Meningitis*  PNA < 7 days: 50 mg/kg q12h  PNA 7-28 days: 50 mg/kg q8h  PNA > 28 days: 50 mg/kg q6h or q4h |  |
|  | Bijleveld *et al*. 2018^14^ | 41 | 40 (36-42) | 5 (2-5) | 3.42 (2.19-4.55) | Majority received either 150.000 IU/kg/day q8h (56%) or 200.000 IU/kg/day q6h (32%) | N=398, 0-60 h after administration |
|  | Muller *et al*. 2007^15^ | 20 | Mean 29.71 (26.43-32) | NA | Mean 1.195  (0.65, 2.03)^a^ | 50.000 IU/kg q12h | N=167, 0-12 h after administration |
|  | Padari *et al*. 2018^16^ | 35 | ≤28 weeks (N=18) | <72 hours | <1.2 | 25.000 or 50.000 IU/kg q12h | N=175^b^ , 0-12 h after administration |
|  |  |  | 32-34 weeks (N=7) | 3 (2-3.5) | 2.1 (2-2.5) |  |  |
|  |  |  | ≥35 weeks (N=10) | 2.5 (2-3) | 3.3 (3-3.9) |  |  |

Values are reported as medians and ranges, unless stated otherwise.

IQR, interquartile range; NR, not reported; PNA postnatal age.

^a^ Current weight, as birth weight was not reported.

^b^ Assumed number of observations based on the sampling schedule and number of patients, as the exact number of observations was not reported.

**Table S4**. Overview of model specification and parameter estimates of evaluated population pharmacokinetic studies.

| **Drug** | **Study** | **Model specification** | **Parameter estimate (RSE%)** |
| --- | --- | --- | --- |
| Amoxicillin | Barker *et al*. 2023^2^ | CL = CL_pop_ × (BW/70)^0.75^ × (PMA^Hill^/(PMA^Hill^+T_50_^Hill^)) × (M+(1-M) × (1−e^(−PNA × N)^)) × (CREAT/(−2.37−12.9 × ln(AGE)+23.9 × AGE^0.5^))^−0.302^ × e^BSVCL^  V = V_pop_ × (BW/70) × e^BSVV^ | CL_pop_ = 16.4 L/h^a^ (7.1)  V_pop_ = 46.2 L^a^ (4.8)  Hill = 2.68 (6.3)  T_50_ = 42.6 (9.1)  M = 0.516 (13.1)  N = 0.020 (50.5)  BSVCL = 16.7% (26.4)  BSVV = 4.5% (57.3)  RUV_proportional_ = 13.5% (16.4) |
|  | Bijleveld *et al*. 2018^10^ | CL = CL_pop_ × (BW/70)^0.75^ × (PNA/2.35)^0.22^ × (TEMP/33.5)^2.43^ × (URINE/2.99)^0.08^ × (GA/40)^3.86^ × e^BSVCL^  Vc = Vc_pop_ × (BW/70) × e^BSVVc^  Q = Q_pop_ × (BW/70)^0.75^  Vp = Vp_pop_ × (BW/70) | CL_pop_ = 2.92 L/h^a^ (4)  Vc_pop_ = 24.1 L^a^ (4)  Q_pop_ = 7.93 L/h^a^ (12)  Vp_pop_ = 24.1 L^a^ (4)  BSVCL = 41.9% (9)  BSVV_c_ = 114.6% (11)  RUV_additive_ = 0.20 mg/L (5) |
|  | Charles *et al*. 1997^11^ | CL = CL_pop_ × (BW x 1000) × e^BSVCL^  V = V_pop_ × e^BSVV^ | CL_pop_ = 0.0000610 L/h (8.1)  V_pop_ = 0.678 L (7.6)  BSVCL = 3.51% (30.1)^c^  BSVV = 36.5% (32.7)^c^  RUV_additive_ = 13.7 mg/L (18.1) |
|  | Keij *et al*. 2023^12^ | CL = CL_pop_ × (BW/70)^0.75^ × (PNA/6.8)^0.357^ × (GA/35.8)^2.37^ × e^BSVCL^  V = V_pop_ × (BW/70) | CL_pop_ = 3.22 L/h^a^ (3)  V_pop_ = 43 L^a^ (2)  BSVCL = 26.7% (NR)  RUV_additive_ = 4.48 mg/L (12)  RUV_proportional_ = 13.2% (10) |
|  | Tang *et al*. 2019^13^ | CL = CL_pop_ × (CW/3.21)^0.75^ × (PNA/7)^0.28^ × (GA/38.14)^4.19^ × e^BSVCL^  Vc = Vc_pop_ × (CW/3.21)  Q = Q_pop_ × (CW/3.21)^0.75^  Vp = Vp_pop_ × (CW/3.21) × e^BSVVp^ | CL_pop_ = 0.81 L/h^b^ (7.5)  Vc_pop_ = 1.48 L^b^ (7.8)  Q_pop_ = 0.17 L/h^b^ (23.7)  Vp_pop_ = 2.42 L^b^ (28.3)  BSVCL = 40.0% (33.5)  BSVV_p_ = 80.0% (55.0)  RUV_proportional_ = 35.0% (13.6) |
| Benzylpenicillin | Barker *et al*. 2023^2^ | CL = CL_pop_ × (BW/70)^0.75^ × (PMA^Hill^/(PMA^Hill^+T_50_^Hill^)) × (M+(1-M) × (1−e^(−PNA × N)^)) × (CREAT/(−2.37−12.91 × ln(AGE)+23.94 × AGE^0.5^))^−0.302^ × e^BSVCL^  V = V_pop_ × (BW/70)^1^ | CL_pop_ = 7.17 L/h^a^ (13.0)  V_pop_ = 11.8 L^a^ (10.5)  T_50_ = 42.6 (9.1)  Hill = 2.68 (6.3)  M = 0.516 (13.1)  N = 0.020 (50.5)  BSVCL = 24.9% (25.6)  RUV_additive_ = 1.2 mg/L (163.1)  RUV_proportional_ = 24.1% (15.5) |
|  | Bijleveld *et al*. 2018^14^ | CL = CL_pop_ × (BW/70)^0.75^ × (PNA/2.21)^0.37^ × (TEMP/33.5)^2.58^ × (0.61)^MOF^ × (GA/40.14)^5.83^ × e^BSVCL^  Vc = Vc_pop_ × (BW/70)^1^ × e^BSVVc^  Q = Q_pop_ × (BW/70)^0.75^  Vp = Vp_pop_ × (BW/70)^1^ | CL_pop_ = 6.69 L/h^a^ (9)  Vc_pop_ = 41.6 L^a^ (8)  Q_pop_ = 0.97 L/h^a^ (20)  Vp_pop_ = 70.9 L^a^ (37)  BSVCL = 45% (15)  BSVVc = 22% (39)  Cov_BSVC, BSVVc_ = 0.08316  RUV_additive_ = 0.34 mg/L (8) |
|  | Muller et al. 2007^15^ | CL = CL_pop_ × e^BSVCL^  Vc = Vc_pop_ × e^BSVVc^  Q = Q_pop_  Vp = Vp_pop_ | CL_pop_ = 0.103 L/h (10.1)  Vc_pop_ = 0.359 L (15.5)  Q_pop_ = 0.774 L/h (35.8)  Vp_pop_ = 0.152 L (20.5)  BSVCL = 0.164 (52.7)  BSVVc = 0.39 (32.3)  RUV_additive_ = 1.12 (79.6)  RUV_proportional_ = 0.104 (30.4) |
|  | Padari et al. 2018^16^ | CL = CL_pop_ × (BW/70)^0.632^×(PMA^Hill^/(PMA^Hill^+T_50_^Hill^))× e^BSVCL^  Vc = Vc_pop_ × (BW/70)^1^ × e^BSVVc^  Q = Q_pop_ × (BW/70)^0.75^ × e^BSVQ^  Vp = Vp_pop_× (BW/70)^1^ × e^BSVVp^ | CL_pop_ = 13.2 L/h^a^ (7.9)  Vc_pop_ = 10.3 L^a^ (21.0)  Q_pop_ = 55.6 L/h^a^ (18.4)  Vp_pop_ = 29.8 L^a^ (8.6)  T_50_ = 55.4 (NR)^d^  Hill = 3.33 (NR)^d^  BSVCL = 39% (NR)  BSVVc = 23% (NR)  BSVVp = 35% (NR)  Cov_BSVCL, BSVVc_ = 0.01  RUV_additive_ = 0.278 mg/L (NR)  RUV_proportional_ = 13% (NR) |

AGE, postnatal age (years); BSV, between-subject variability (reported as variance unless otherwise specified); BW, bodyweight at birth (kg); CL, clearance; Cov, covariance; CREAT, serum creatinine (μmol/L); CW, current bodyweight (kg); GA, gestational age (weeks); Hill, Hill coefficient; M, fraction of clearance on day of birth; N, rate of clearance maturation after birth; PMA, postmenstrual age (weeks); PNA, postnatal age (days); POP, population; Q, intercompartmental clearance; RSE, relative standard error; RUV, residual unexplained variability; T_50_ , clearance maturation half time (PMA in weeks); TEMP, body temperature (°C); URINE, urine output (ml/kg/h); Vc, central distribution volume; Vp, peripheral distribution volume.

*a* allometrically scaled to a total bodyweight of 70 kg.

*b* allometrically scaled to a total bodyweight of 3.21 kg.

*c* between-subject variability reported as standard deviation.

*d* not reported in the paper, but obtained through personal communication with the authors.

**Table S5.** Numerical predictive performance metrics for all models.

| **Drug** | **Model** | **Mean PPE**  [95% CI]  (%) | **Mean APPE**  [95% CI]  (%) | **NRMSE**  (%) | **P_10_**  (%) | **P_20_**  (%) | **P_30_**  (%) |
| --- | --- | --- | --- | --- | --- | --- | --- |
| Amoxicillin | Barker | 29.1 [24.7; 33.5] | 36.4 [33.4; 39.3] | 49.9 | 5.3 | 14.7 | 34.7 |
|  | Bijleveld | -20.6 [-28.5; -12.8] | 35.1 [28.6; 41.7] | 33.1 | 27.1 | 48.8 | 65.3 |
|  | Charles | -14.4 [-23.4; -5.32] | 41.2 [34.3; 48.1] | 62.6 | 18.2 | 34.7 | 55.3 |
|  | Keij | -35.8 [-45.7; -25.9] | 50.0 [41.7; 58.4] | 40.2 | 17.6 | 36.5 | 50.0 |
|  | Tang | 26.1 [21.4; 30.9] | 32.5 [28.8; 36.3] | 44.0 | 24.1 | 39.4 | 54.1 |
| Benzylpenicillin | Barker | -162.39 [-196.68; -128.10] | 167.29 [134.18; 200.40] | 146.04 | 2.4 | 4.1 | 5.9 |
|  | Bijleveld | -99.14 [-153.77; -44.50] | 134.44 [83.62; 185.25] | 81.68 | 3.5 | 12.4 | 16.5 |
|  | Muller | -186.35 [-264.75; -107.95] | 203.41 [127.12; 279.71] | 156.24 | 2.9 | 5.9 | 9.4 |
|  | Padari | -16.31 [-34.81; -2.20] | 55.71 [41.44; 69.98] | 76.01 | 4.7 | 11.2 | 22.4 |

APPE, absolute percentage prediction error; NRMSE, normalized root mean squared error; P_10_, percentage of population-predicted concentrations falling within 10% of observed concentrations; P_20_, percentage of population-predicted concentrations falling within 20% of observed concentrations; P_30_, percentage of population-predicted concentrations falling within 30% of observed concentrations;

PPE, percentage prediction error.

**Table S6.** Parameter estimates of the original Padari model and after between-subject variability re-estimation.

| **Parameter** | **Original Padari model** | **Padari model with BSV re-estimation** |
| --- | --- | --- |
|  | *Median estimate* | *Median estimate* |
| CL | 13.2^a^ | 13.2^a^ |
| *CL BSV (%) [shrinkage; %]* | 39^a^ | 32.2 [39.6] |
| *BW effect on CL* | 0.632^a^ | 0.632^a^ |
| Vc | 10.3^a^ | 10.3^a^ |
| *Vc BSV (%) [shrinkage; %]* | 23^a^ | 15.1 [54.78] |
| *BW effect on Vc* | 1^a^ | 1^a^ |
| Vp (BSV%) [shrinkage%] | 29.8^a^ | 29.8^a^ |
| *Vp BSV (%) [shrinkage; %]* | 35^a^ | 29.9 [53.9] |
| *BW effect on Vp* | 1^a^ | 1^a^ |
| Q | 55.6^a^ | 55.6^a^ |
| *BW effect on Q* | 0.75^a^ | 0.75^a^ |
| Hill | 3.33^a^ | 3.33^a^ |
| T_50_ | 55.4^a^ | 55.4^a^ |
| Additive error | 0.278^a^ | 0.676^a^ |
| Proportional error | 0.13^a^ | 0.559^a^ |
| **OFV** | **780.85** | **443.36** |

BSV, between-subject variability; BW, bodyweight (in kg); CL, clearance; Hill, Hill coefficient; OFV, objective function value; Q, intercompartmental clearance; T_50_, clearance maturation half time (PMA in weeks); Vc, central distribution volume; Vp, peripheral distribution volume.

*a* parameter estimate fixed to the value from the original Padari model

**Table S7.** PTA for amoxicillin and benzylpenicillin for all evaluated neonatal formularies, MIC breakpoints, and pharmacokinetic/pharmacodynamic (PK/PD) targets.

| **Formulary** | **PK/PD target** | **Amoxicillin**  *PTA for each MIC breakpoint* (%) | | | **Benzylpenicillin**  *PTA for each MIC breakpoint* (%) | | |
| --- | --- | --- | --- | --- | --- | --- | --- |
|  |  | *0.125 mg/L* | *0.25 mg/L* | *1 mg/L* | *0.125 mg/L* | *0.25 mg/L* | *1 mg/L* |
| ANMF | 40%fT>MIC | 100 | 100 | 100 | 100 | 100 | 100 |
|  | 50%fT>MIC | 100 | 100 | 100 | 100 | 100 | 99.7 |
|  | 60%fT>MIC | 100 | 100 | 100 | 100 | 99.9 | 99.0 |
|  | 70%fT>MIC | 100 | 100 | 100 | 99.8 | 99.6 | 97.6 |
|  | 80%fT>MIC | 100 | 100 | 100 | 99.6 | 99.1 | 95.5 |
|  | 90%fT>MIC | 100 | 100 | 100 | 99.1 | 98.2 | 92.9 |
|  | 100%fT>MIC | 100 | 100 | 99.9 | 98.2 | 96.6 | 88.8 |
|  | 100%fT>4×MIC | 100 | 99.9 | 97.6 | 94.0 | 88.8 | 62.2 |
| BNFC | 40%fT>MIC | 100 | 100 | 100 | 100 | 100 | 99.4 |
|  | 50%fT>MIC | 100 | 100 | 100 | 100 | 99.8 | 98.3 |
|  | 60%fT>MIC | 100 | 100 | 100 | 99.7 | 99.4 | 96.2 |
|  | 70%fT>MIC | 100 | 100 | 100 | 99.3 | 98.6 | 92.8 |
|  | 80%fT>MIC | 100 | 100 | 100 | 98.6 | 97.4 | 88.3 |
|  | 90%fT>MIC | 100 | 100 | 100 | 97.6 | 95.8 | 82.5 |
|  | 100%fT>MIC | 100 | 100 | 99.9 | 96.1 | 92.8 | 75.4 |
|  | 100%fT>4×MIC | 100 | 99.9 | 98.3 | 86.8 | 75.4 | 28.5 |
| DPF | 40%fT>MIC | 100 | 100 | 100 | 100 | 100 | 99.8 |
|  | 50%fT>MIC | 100 | 100 | 100 | 100 | 100 | 98.9 |
|  | 60%fT>MIC | 100 | 100 | 100 | 100 | 99.9 | 97.4 |
|  | 70%fT>MIC | 100 | 100 | 100 | 99.9 | 99.5 | 94.6 |
|  | 80%fT>MIC | 100 | 100 | 100 | 99.6 | 98.8 | 90.1 |
|  | 90%fT>MIC | 100 | 100 | 100 | 99.1 | 97.7 | 85.4 |
|  | 100%fT>MIC | 100 | 100 | 100 | 98.1 | 95.8 | 78.0 |
|  | 100%fT>4×MIC | 100 | 100 | 97.2 | 90.4 | 78.0 | 16.5 |
| Lexicomp | 40%fT>MIC | NA | NA | NA | 100 | 100 | 99.8 |
|  | 50%fT>MIC | NA | NA | NA | 100 | 99.9 | 98.9 |
|  | 60%fT>MIC | NA | NA | NA | 99.8 | 99.6 | 97.0 |
|  | 70%fT>MIC | NA | NA | NA | 99.6 | 99.0 | 94.2 |
|  | 80%fT>MIC | NA | NA | NA | 99.0 | 97.9 | 89.9 |
|  | 90%fT>MIC | NA | NA | NA | 98.1 | 96.2 | 84.8 |
|  | 100%fT>MIC | NA | NA | NA | 96.4 | 93.8 | 78.5 |
|  | 100%fT>4×MIC | NA | NA | NA | 88.1 | 78.5 | 35.9 |
| NDPGH | 40%fT>MIC | NA | NA | NA | 100 | 100 | 98.8 |
|  | 50%fT>MIC | NA | NA | NA | 99.9 | 99.7 | 96.0 |
|  | 60%fT>MIC | NA | NA | NA | 99.6 | 99.1 | 91.8 |
|  | 70%fT>MIC | NA | NA | NA | 99.1 | 97.7 | 86.0 |
|  | 80%fT>MIC | NA | NA | NA | 98.0 | 95.6 | 79.4 |
|  | 90%fT>MIC | NA | NA | NA | 96.3 | 92.8 | 72.2 |
|  | 100%fT>MIC | NA | NA | NA | 93.8 | 89.1 | 62.8 |
|  | 100%fT>4×MIC | NA | NA | NA | 79.4 | 62.8 | 10.2 |
| NeoFax | 40%fT>MIC | NA | NA | NA | 100 | 100 | 99.8 |
|  | 50%fT>MIC | NA | NA | NA | 100 | 100 | 98.9 |
|  | 60%fT>MIC | NA | NA | NA | 99.9 | 99.6 | 97.2 |
|  | 70%fT>MIC | NA | NA | NA | 99.6 | 99.0 | 94.5 |
|  | 80%fT>MIC | NA | NA | NA | 99.0 | 97.9 | 90.6 |
|  | 90%fT>MIC | NA | NA | NA | 98.2 | 96.5 | 85.9 |
|  | 100%fT>MIC | NA | NA | NA | 96.8 | 94.1 | 79.3 |
|  | 100%fT>4×MIC | NA | NA | NA | 88.9 | 79.3 | 36.6 |
| NF | 40%fT>MIC | 100 | 100 | 100 | 100 | 100 | 99.8 |
|  | 50%fT>MIC | 100 | 100 | 100 | 100 | 99.9 | 98.9 |
|  | 60%fT>MIC | 100 | 100 | 100 | 99.9 | 99.6 | 97.1 |
|  | 70%fT>MIC | 100 | 100 | 100 | 99.6 | 99.0 | 94.3 |
|  | 80%fT>MIC | 100 | 100 | 100 | 99.0 | 98.0 | 90.4 |
|  | 90%fT>MIC | 100 | 100 | 100 | 98.2 | 96.4 | 85.6 |
|  | 100%fT>MIC | 100 | 100 | 99.9 | 96.7 | 94.0 | 78.4 |
|  | 100%fT>4×MIC | 100 | 99.9 | 97.6 | 88.7 | 78.4 | 35.8 |
| SPD | 40%fT>MIC | 100 | 100 | 100 | 100 | 100 | 99.7 |
|  | 50%fT>MIC | 100 | 100 | 100 | 100 | 99.9 | 99.0 |
|  | 60%fT>MIC | 100 | 100 | 100 | 99.8 | 99.5 | 97.1 |
|  | 70%fT>MIC | 100 | 100 | 100 | 99.5 | 99.0 | 94.1 |
|  | 80%fT>MIC | 100 | 100 | 100 | 99.1 | 97.9 | 90.2 |
|  | 90%fT>MIC | 100 | 100 | 100 | 98.1 | 96.3 | 85.2 |
|  | 100%fT>MIC | 100 | 100 | 99.9 | 96.7 | 93.7 | 78.6 |
|  | 100%fT>4×MIC | 100 | 99.9 | 97.6 | 88.3 | 78.6 | 35.6 |

ANMF, Australasian Neonatal Medicines Formulary; BNFC, British National Formulary for Children; DPF, Dutch/German/Austrian/Norwegian Pediatric Formularies; Lexicomp, Pediatric and Neonatal Lexi-Drugs; NDPGH, Neonatal Dosage and Practical Guidelines Handbook; NF, Neonatal Formulary; SPD, SwissPedDose.

**Table S8.** Sensitivity analysis for the influence of protein binding on the PTA results, for each drug, neonatal formulary, pharmacokinetic/pharmacodynamic (PK/PD) target and MIC.

| **Formulary** | **PK/PD target** | **PTA (%) for each drug, MIC breakpoint and degree of protein binding (%)** | | | | | | | | | | | | | | | | | |
| --- | --- | --- | --- | --- | --- | --- | --- | --- | --- | --- | --- | --- | --- | --- | --- | --- | --- | --- | --- |
|  |  | *Amoxicillin* | | | | | | | | | *Benzylpenicillin* | | | | | | | | |
|  |  | 0.125 mg/L | | | 0.25 mg/L | | | 1 mg/L | | | 0.125 mg/L | | | 0.25 mg/L | | | 1 mg/L | | |
|  |  | PB5 | PB10 | PB15 | PB5 | PB10 | PB15 | PB5 | PB10 | PB15 | PB40 | PB50 | PB60 | PB40 | PB50 | PB60 | PB40 | PB50 | PB60 |
| ANMF | 40%fT>MIC | 100 | 100 | 100 | 100 | 100 | 100 | 100 | 100 | 100 | 100 | 100 | 100 | 100 | 100 | 100 | 100 | 99.9 | 99.9 |
|  | 50%fT>MIC | 100 | 100 | 100 | 100 | 100 | 100 | 100 | 100 | 100 | 100 | 100 | 100 | 100 | 100 | 99.9 | 100 | 99.5 | 99.4 |
|  | 60%fT>MIC | 100 | 100 | 100 | 100 | 100 | 100 | 100 | 100 | 100 | 100 | 99.9 | 99.9 | 99.8 | 99.8 | 99.7 | 99.0 | 98.8 | 98.3 |
|  | 70%fT>MIC | 100 | 100 | 100 | 100 | 100 | 100 | 100 | 100 | 100 | 99.8 | 99.7 | 99.6 | 99.5 | 99.4 | 99.2 | 97.8 | 97.4 | 96.6 |
|  | 80%fT>MIC | 100 | 100 | 100 | 100 | 100 | 100 | 100 | 100 | 100 | 99.5 | 99.4 | 99.2 | 99.0 | 98.8 | 98.5 | 96.2 | 95.3 | 94.1 |
|  | 90%fT>MIC | 100 | 100 | 100 | 100 | 100 | 100 | 100 | 100 | 100 | 99.0 | 98.9 | 98.6 | 98.2 | 97.9 | 97.4 | 94.0 | 92.8 | 90.9 |
|  | 100%fT>MIC | 100 | 100 | 100 | 100 | 100 | 100 | 99.9 | 99.9 | 99.9 | 98.1 | 97.9 | 97.5 | 96.9 | 96.3 | 95.6 | 90.4 | 88.6 | 85.9 |
|  | 100%fT>4×MIC | 100 | 100 | 100 | 99.9 | 99.9 | 99.9 | 97.9 | 97.7 | 97.4 | 94.6 | 93.8 | 92.7 | 90.4 | 88.6 | 85.9 | 67.5 | 61.8 | 54.4 |
| BNFC | 40%fT>MIC | 100 | 100 | 100 | 100 | 100 | 100 | 100 | 100 | 100 | 100 | 100 | 100 | 100 | 100 | 100 | 99.6 | 99.4 | 99.2 |
|  | 50%fT>MIC | 100 | 100 | 100 | 100 | 100 | 100 | 100 | 100 | 100 | 100 | 100 | 99.9 | 99.9 | 99.8 | 99.7 | 98.8 | 98.2 | 97.5 |
|  | 60%fT>MIC | 100 | 100 | 100 | 100 | 100 | 100 | 100 | 100 | 100 | 99.8 | 99.7 | 99.6 | 99.5 | 99.4 | 99.2 | 96.8 | 95.8 | 94.4 |
|  | 70%fT>MIC | 100 | 100 | 100 | 100 | 100 | 100 | 100 | 100 | 100 | 99.4 | 99.4 | 99.2 | 98.9 | 98.6 | 98.2 | 94.0 | 92.2 | 89.8 |
|  | 80%fT>MIC | 100 | 100 | 100 | 100 | 100 | 100 | 100 | 100 | 100 | 98.9 | 98.6 | 98.3 | 97.7 | 97.3 | 96.5 | 89.8 | 87.5 | 84.2 |
|  | 90%fT>MIC | 100 | 100 | 100 | 100 | 100 | 100 | 100 | 100 | 100 | 97.9 | 97.6 | 97.1 | 96.1 | 95.4 | 94.3 | 85.0 | 82.3 | 78.0 |
|  | 100%fT>MIC | 100 | 100 | 100 | 100 | 100 | 100 | 99.9 | 99.9 | 99.9 | 96.4 | 95.8 | 94.9 | 93.5 | 92.5 | 90.9 | 78.5 | 75.2 | 70.2 |
|  | 100%fT>4×MIC | 100 | 100 | 100 | 99.9 | 99.9 | 99.9 | 98.6 | 98.4 | 98.2 | 88.1 | 86.1 | 83.3 | 78.5 | 75.2 | 70.2 | 36.1 | 28.6 | 19.7 |
| DPF | 40%fT>MIC | 100 | 100 | 100 | 100 | 100 | 100 | 100 | 100 | 100 | 100 | 100 | 100 | 100 | 100 | 100 | 99.9 | 99.8 | 99.5 |
|  | 50%fT>MIC | 100 | 100 | 100 | 100 | 100 | 100 | 100 | 100 | 100 | 100 | 100 | 100 | 100 | 100 | 99.9 | 99.4 | 99.0 | 98.3 |
|  | 60%fT>MIC | 100 | 100 | 100 | 100 | 100 | 100 | 100 | 100 | 100 | 100 | 99.9 | 99.9 | 99.9 | 99.9 | 99.8 | 98.2 | 97.3 | 95.5 |
|  | 70% fT>MIC | 100 | 100 | 100 | 100 | 100 | 100 | 100 | 100 | 100 | 99.9 | 99.9 | 99.8 | 99.6 | 99.5 | 99.3 | 95.9 | 94.2 | 91.8 |
|  | 80%fT>MIC | 100 | 100 | 100 | 100 | 100 | 100 | 100 | 100 | 100 | 99.7 | 99.6 | 99.5 | 99.1 | 98.9 | 98.3 | 92.8 | 90.5 | 86.9 |
|  | 90%fT>MIC | 100 | 100 | 100 | 100 | 100 | 100 | 100 | 100 | 100 | 99.3 | 99.1 | 98.8 | 98.2 | 97.8 | 96.9 | 88.8 | 85.8 | 81.2 |
|  | 100%fT>MIC | 100 | 100 | 100 | 100 | 100 | 100 | 100 | 100 | 99.9 | 98.6 | 98.2 | 97.6 | 96.8 | 95.9 | 94.7 | 82.7 | 78.2 | 71.2 |
|  | 100%fT>4×MIC | 100 | 100 | 100 | 100 | 100 | 99.9 | 97.8 | 97.4 | 96.9 | 92.3 | 90.4 | 87.8 | 82.7 | 78.2 | 71.2 | 24.6 | 16.1 | 7.8 |
| Lexicomp | 40%fT>MIC | NA | NA | NA | NA | NA | NA | NA | NA | NA | 100 | 100 | 100 | 100 | 100 | 100 | 99.7 | 99.7 | 99.5 |
|  | 50%fT>MIC | NA | NA | NA | NA | NA | NA | NA | NA | NA | 100 | 99.9 | 99.9 | 99.9 | 99.8 | 99.8 | 99.2 | 99.0 | 98.5 |
|  | 60%fT>MIC | NA | NA | NA | NA | NA | NA | NA | NA | NA | 99.8 | 99.8 | 99.8 | 99.7 | 99.5 | 99.5 | 98.0 | 97.4 | 96.2 |
|  | 70% fT>MIC | NA | NA | NA | NA | NA | NA | NA | NA | NA | 99.6 | 99.5 | 99.4 | 99.2 | 99.0 | 98.8 | 95.6 | 94.2 | 92.0 |
|  | 80%fT>MIC | NA | NA | NA | NA | NA | NA | NA | NA | NA | 99.2 | 99.1 | 98.8 | 98.4 | 98.1 | 97.6 | 91.8 | 90.0 | 87.0 |
|  | 90%fT>MIC | NA | NA | NA | NA | NA | NA | NA | NA | NA | 98.5 | 98.3 | 97.9 | 97.3 | 96.6 | 95.6 | 87.4 | 85.1 | 81.6 |
|  | 100%fT>MIC | NA | NA | NA | NA | NA | NA | NA | NA | NA | 97.4 | 97.0 | 96.1 | 94.6 | 93.7 | 92.2 | 81.9 | 78.7 | 74.3 |
|  | 100%fT>4×MIC | NA | NA | NA | NA | NA | NA | NA | NA | NA | 90.0 | 88.1 | 85.7 | 81.9 | 78.7 | 74.3 | 43.2 | 36.1 | 26.3 |
| NDPGH | 40%fT>MIC | NA | NA | NA | NA | NA | NA | NA | NA | NA | 100 | 100 | 100 | 99.9 | 99.9 | 99.9 | 98.9 | 98.5 | 97.7 |
|  | 50%fT>MIC | NA | NA | NA | NA | NA | NA | NA | NA | NA | 99.9 | 99.9 | 99.8 | 99.7 | 99.6 | 99.4 | 97.0 | 96.0 | 93.8 |
|  | 60%fT>MIC | NA | NA | NA | NA | NA | NA | NA | NA | NA | 99.6 | 99.5 | 99.3 | 99.0 | 98.8 | 98.4 | 93.4 | 91.3 | 88.0 |
|  | 70% fT>MIC | NA | NA | NA | NA | NA | NA | NA | NA | NA | 99.0 | 98.8 | 98.5 | 98.0 | 97.4 | 96.8 | 88.4 | 85.8 | 81.0 |
|  | 80%fT>MIC | NA | NA | NA | NA | NA | NA | NA | NA | NA | 98.2 | 97.8 | 97.3 | 96.4 | 95.4 | 93.9 | 82.8 | 78.8 | 73.0 |
|  | 90%fT>MIC | NA | NA | NA | NA | NA | NA | NA | NA | NA | 96.8 | 96.2 | 95.2 | 93.6 | 92.3 | 90.5 | 75.8 | 71.3 | 64.7 |
|  | 100%fT>MIC | NA | NA | NA | NA | NA | NA | NA | NA | NA | 94.5 | 93.4 | 92.2 | 90.0 | 88.4 | 86.0 | 66.7 | 61.1 | 53.1 |
|  | 100%fT>4×MIC | NA | NA | NA | NA | NA | NA | NA | NA | NA | 82.2 | 78.9 | 74.1 | 66.7 | 61.1 | 53.1 | 15.0 | 9.5 | 4.4 |
| NeoFax | 40%fT>MIC | NA | NA | NA | NA | NA | NA | NA | NA | NA | 100 | 100 | 100 | 100 | 100 | 100 | 100 | 99.8 | 99.6 |
|  | 50%fT>MIC | NA | NA | NA | NA | NA | NA | NA | NA | NA | 100 | 100 | 100 | 99.9 | 99.9 | 99.9 | 99.1 | 98.8 | 98.1 |
|  | 60%fT>MIC | NA | NA | NA | NA | NA | NA | NA | NA | NA | 99.9 | 99.9 | 99.9 | 99.8 | 99.7 | 99.5 | 97.5 | 96.9 | 95.7 |
|  | 70% fT>MIC | NA | NA | NA | NA | NA | NA | NA | NA | NA | 99.7 | 99.6 | 99.4 | 99.2 | 98.9 | 98.5 | 95.1 | 93.7 | 91.8 |
|  | 80%fT>MIC | NA | NA | NA | NA | NA | NA | NA | NA | NA | 99.2 | 98.9 | 98.6 | 98.1 | 97.7 | 97.1 | 91.6 | 89.8 | 86.8 |
|  | 90%fT>MIC | NA | NA | NA | NA | NA | NA | NA | NA | NA | 98.2 | 98.0 | 97.5 | 96.7 | 96.0 | 95.0 | 87.2 | 84.6 | 81.3 |
|  | 100%fT>MIC | NA | NA | NA | NA | NA | NA | NA | NA | NA | 96.8 | 96.4 | 95.5 | 94.2 | 93.3 | 92.0 | 81.5 | 78.5 | 74.0 |
|  | 100%fT>4×MIC | NA | NA | NA | NA | NA | NA | NA | NA | NA | 89.7 | 87.9 | 85.3 | 81.5 | 78.5 | 74.0 | 43.0 | 35.4 | 26.0 |
| NF | 40%fT>MIC | 100 | 100 | 100 | 100 | 100 | 100 | 100 | 100 | 100 | 100 | 100 | 100 | 100 | 100 | 100 | 99.7 | 99.6 | 99.4 |
|  | 50%fT>MIC | 100 | 100 | 100 | 100 | 100 | 100 | 100 | 100 | 100 | 100 | 100 | 99.9 | 99.9 | 99.8 | 99.7 | 99.1 | 98.8 | 98.2 |
|  | 60%fT>MIC | 100 | 100 | 100 | 100 | 100 | 100 | 100 | 100 | 100 | 99.8 | 99.8 | 99.7 | 99.5 | 99.4 | 99.3 | 97.5 | 96.8 | 95.7 |
|  | 70%fT>MIC | 100 | 100 | 100 | 100 | 100 | 100 | 100 | 100 | 100 | 99.4 | 99.4 | 99.3 | 99.1 | 98.8 | 98.5 | 95.2 | 93.9 | 91.9 |
|  | 80%fT>MIC | 100 | 100 | 100 | 100 | 100 | 100 | 100 | 100 | 100 | 99.0 | 98.9 | 98.6 | 98.1 | 97.7 | 97.1 | 91.7 | 89.8 | 86.9 |
|  | 90%fT>MIC | 100 | 100 | 100 | 100 | 100 | 100 | 100 | 100 | 100 | 98.3 | 98.0 | 97.5 | 96.7 | 96.1 | 95.2 | 87.4 | 84.7 | 80.9 |
|  | 100%fT>MIC | 100 | 100 | 100 | 100 | 100 | 100 | 99.9 | 99.9 | 99.9 | 96.9 | 96.4 | 95.6 | 94.2 | 93.4 | 91.9 | 81.0 | 77.8 | 73.1 |
|  | 100%fT>4×MIC | 100 | 100 | 100 | 99.9 | 99.9 | 99.9 | 97.9 | 97.7 | 97.4 | 90.0 | 88.1 | 85.5 | 81.0 | 77.8 | 73.1 | 43.0 | 35.4 | 25.7 |
| SPD | 40%fT>MIC | 100 | 100 | 100 | 100 | 100 | 100 | 100 | 100 | 100 | 100 | 100 | 100 | 100 | 100 | 100 | 99.8 | 99.7 | 99.5 |
|  | 50%fT>MIC | 100 | 100 | 100 | 100 | 100 | 100 | 100 | 100 | 100 | 100 | 99.9 | 99.9 | 99.9 | 99.9 | 99.8 | 99.1 | 98.7 | 98.2 |
|  | 60%fT>MIC | 100 | 100 | 100 | 100 | 100 | 100 | 100 | 100 | 100 | 99.9 | 99.8 | 99.7 | 99.6 | 99.5 | 99.3 | 97.6 | 96.9 | 95.5 |
|  | 70%fT>MIC | 100 | 100 | 100 | 100 | 100 | 100 | 100 | 100 | 100 | 99.6 | 99.5 | 99.3 | 99.0 | 98.9 | 98.6 | 94.9 | 93.6 | 91.6 |
|  | 80%fT>MIC | 100 | 100 | 100 | 100 | 100 | 100 | 100 | 100 | 100 | 99.0 | 98.9 | 98.7 | 98.2 | 97.7 | 97.1 | 91.4 | 89.8 | 87.0 |
|  | 90%fT>MIC | 100 | 100 | 100 | 100 | 100 | 100 | 100 | 100 | 100 | 98.3 | 98.0 | 97.4 | 96.5 | 95.9 | 94.8 | 87.5 | 85.2 | 81.5 |
|  | 100%fT>MIC | 100 | 100 | 100 | 100 | 100 | 100 | 99.9 | 99.9 | 99.9 | 96.8 | 96.2 | 95.3 | 94.2 | 93.4 | 91.8 | 81.7 | 78.8 | 74.4 |
|  | 100%fT>4×MIC | 100 | 100 | 100 | 99.9 | 99.9 | 99.9 | 97.9 | 97.7 | 97.4 | 89.8 | 88.2 | 86.1 | 81.7 | 78.8 | 74.4 | 43.2 | 35.6 | 26.7 |

ANMF, Australasian Neonatal Medicines Formulary; PB5-PB60, 5-60% protein binding; BNFC, British National Formulary for Children; DPF, Dutch/German/Austrian/Norwegian Pediatric Formularies; NF, Neonatal Formulary; SPD, SwissPedDose; %fT>MIC, percentage of time that the unbound (free) drug concentration exceeds the MIC.

**Table S9.** PTA for benzylpenicillin with both proposed alternative dosing regimens, for all MIC breakpoints, and pharmacokinetic/pharmacodynamic (PK/PD) targets.

| **Formulary** | **PK/PD target** | **Benzylpenicillin**  *PTA for each MIC breakpoint* (%) | | |
| --- | --- | --- | --- | --- |
|  |  | *0.125 mg/L* | *0.25 mg/L* | *1 mg/L* |
| Continuous | 40%fT>MIC | 100 | 100 | 100 |
|  | 50%fT>MIC | 100 | 100 | 100 |
|  | 60%fT>MIC | 100 | 100 | 100 |
|  | 70%fT>MIC | 100 | 100 | 100 |
|  | 80%fT>MIC | 100 | 100 | 100 |
|  | 90%fT>MIC | 100 | 100 | 100 |
|  | 100%fT>MIC | 100 | 100 | 100 |
|  | 100%fT>4×MIC | 100 | 100 | 75.0 |
| Intermittent | 40%fT>MIC | 100 | 100 | 100 |
|  | 50%fT>MIC | 100 | 100 | 99.9 |
|  | 60%fT>MIC | 100 | 100 | 99.6 |
|  | 70%fT>MIC | 100 | 100 | 99.2 |
|  | 80%fT>MIC | 100 | 99.9 | 98.1 |
|  | 90%fT>MIC | 99.9 | 99.6 | 96.5 |
|  | 100%fT>MIC | 99.7 | 99.3 | 94.2 |
|  | 100%fT>4×MIC | 98.0 | 94.2 | 43.9 |

**Table S10.** Sensitivity analysis for the influence of protein binding (PB) on the PTA results for benzylpenicillin with the proposed alternative dosing regimens, for each pharmacokinetic/pharmacodynamic (PK/PD) target and MIC breakpoint.

| **Regimen** | **PK/PD target** | **PTA (%) for each MIC breakpoint and degree of protein binding (%)** | | | | | | | | |
| --- | --- | --- | --- | --- | --- | --- | --- | --- | --- | --- |
|  |  | 0.125 mg/L | | | 0.25 mg/L | | | 1 mg/L | | |
|  |  | PB40 | PB50 | PB60 | PB40 | PB50 | PB60 | PB40 | PB50 | PB60 |
| Continuous | 40%fT>MIC | 100 | 100 | 100 | 100 | 100 | 100 | 100 | 100 | 100 |
|  | 50%fT>MIC | 100 | 100 | 100 | 100 | 100 | 100 | 100 | 100 | 100 |
|  | 60%fT>MIC | 100 | 100 | 100 | 100 | 100 | 100 | 100 | 100 | 100 |
|  | 70%fT>MIC | 100 | 100 | 100 | 100 | 100 | 100 | 100 | 100 | 100 |
|  | 80%fT>MIC | 100 | 100 | 100 | 100 | 100 | 100 | 100 | 100 | 100 |
|  | 90%fT>MIC | 100 | 100 | 100 | 100 | 100 | 100 | 100 | 100 | 100 |
|  | 100%fT>MIC | 100 | 100 | 100 | 100 | 100 | 100 | 100 | 100 | 100 |
|  | 100%fT>4×MIC | 100 | 100 | 100 | 100 | 100 | 100 | 89.4 | 73.4 | 45.9 |
| Intermittent | 40%fT>MIC | 100 | 100 | 100 | 100 | 100 | 100 | 100 | 100 | 100 |
|  | 50%fT>MIC | 100 | 100 | 100 | 100 | 100 | 100 | 100 | 99.9 | 99.9 |
|  | 60%fT>MIC | 100 | 100 | 100 | 100 | 100 | 100 | 99.8 | 99.6 | 99.4 |
|  | 70%fT>MIC | 100 | 100 | 100 | 100 | 100 | 99.9 | 99.3 | 99.1 | 98.5 |
|  | 80%fT>MIC | 100 | 100 | 99.9 | 99.9 | 99.9 | 99.7 | 98.6 | 98.0 | 97.0 |
|  | 90%fT>MIC | 99.9 | 99.9 | 99.9 | 99.7 | 99.6 | 99.5 | 97.4 | 96.4 | 94.8 |
|  | 100%fT>MIC | 99.8 | 99.7 | 99.6 | 99.4 | 99.3 | 99.0 | 95.6 | 94.0 | 91.3 |
|  | 100%fT>4×MIC | 98.5 | 97.9 | 97.2 | 95.6 | 94.0 | 91.3 | 55.0 | 42.8 | 25.5 |

PB40-PB60, 40-60% protein binding; %fT>MIC, percentage of time that the unbound (free) drug concentration exceeds the MIC.

**Supplementary material**

**Bioanalytical validation**

Amoxicillin and benzylpenicillin were quantified in serum using a previously validated LC-MS/MS assay capable of the simultaneous quantification of amoxicillin, benzylpenicillin, meropenem and flucloxacillin in serum,^17^ used for routine amoxicillin and benzylpenicillin TDM at LUMC. A full bioanalytical method validation as per the EMA guidelines on bioanalytical method validation was conducted previously. An overview of its analytical performance for amoxicillin and benzylpenicillin is provided below.

| **Parameter** | **N** | **Amoxicillin** | **Benzylpenicillin** |
| --- | --- | --- | --- |
| Within-day accuracy (%) |  |  |  |
| *Low* | 5 | 106.8 | 100.3 |
| *Medium* | 5 | 103.6 | 102.1 |
| *High* | 5 | 109.8 | 105.7 |
| Between-day accuracy (%) |  |  |  |
| *Low* | 15 | 101.5 | 99.4 |
| *Medium* | 15 | 101.6 | 102.4 |
| *High* | 15 | 107.7 | 105.7 |
| Within-day precision (%CV) |  |  |  |
| *Low* | 5 | 2.2 | 2.3 |
| *Medium* | 5 | 5.3 | 2.6 |
| *High* | 5 | 2.2 | 4.1 |
| Between-day precision (%CV) |  |  |  |
| *Low* | 15 | 4.5 | 2.6 |
| *Medium* | 15 | 3.5 | 1.8 |
| *High* | 15 | 3.1 | 2.5 |
| Linearity (mg/L) |  |  |  |
| *LOQ* | N/A | 0.6 | 0.6 |
| *ULQ* | N/A | 100 | 50 |
| Measurement uncertainty (%) |  |  |  |
| *Low* | N/A | 9.9 | 5.6 |
| *Medium* | N/A | 7.8 | 5.0 |
| *High* | N/A | 13.1 | 9.8 |

**PubMed search queries**

Amoxicillin

(amoxicillin OR augmentin) AND (neonate OR pediatric) AND (pharmacokinetics) AND (model OR NONMEM)

Benzylpenicillin

(benzylpenicillin OR (penicillin G) OR penicillins) AND (neonate OR infant) AND (pharmacokinetics OR (nonlinear mixed effects)) AND (sepsis OR bacterial infection OR Group B Streptococcus OR asphyxia) AND (model OR NONMEM)

***nlmixr2* model code**

Amoxicillin - Barker model

barker_nlmixr_model <- function() {

ini({

tcl <- fix(log(16.4))

tv1 <- fix(log(46.2))

eta.cl ~ fix(0.167)

eta.v1 ~ fix(0.045)

prop.sd <- 0.367

})

model({

tpma <- PMADAYS/7

mf <- (tpma^2.68)/((tpma^2.68) + (42.6^2.68))

clpna <- 0.516 + (1-0.516) * (1-exp(-(PNADAYS/7)*0.020))

scov <- 1

cl <- exp(tcl + eta.cl) * ((BWKG/70)^0.75) * mf * clpna * scov

v1 <- exp(tv1 + eta.v1) * (BWKG/70)

k = cl/v1

d/dt(center) = (-k*center)

cp=center/v1

IPRED = cp

IPRED ~ prop(prop.sd)

})

}

Amoxicillin - Bijleveld model

bijleveld_nlmixr_model <- function() {

ini({

tcl <- fix(log(2.92))

tv1 <- fix(log(24.1))

tq <- fix(log(7.93))

tv2 <- fix(log(24.1))

eta.cl + eta.v1 ~ c(fix(0.419^2), fix(0.23), fix(1.146^2))

add.sd <- 0.20

})

model({

cl <- exp(tcl + eta.cl) * ((BWKG/70)^0.75) * (PNADAYS/2.35)^0.22 * ((GADAYS/280)^3.86) * ((37/33.5)^2.43)

v1 <- exp(tv1 + eta.v1) * (BWKG/70)^1

q <- exp(tq) * ((BWKG/70)^0.75)

v2 <- exp(tv2) * (BWKG/70)^1

k = cl/v1

k12 = q/v1

k21 = q/v2

d/dt(center) = (-k*center) + (k21*periph) - (k12*center)

d/dt(periph) = (k12*center) - (k21*periph)

cp=center/v1

IPRED = cp

IPRED ~ add(add.sd)

})

}

Amoxicillin - Charles model

charles_nlmixr_model <- function() {

ini({

tcl <- fix(log(0.0000610))

tv1 <- fix(log(0.678))

eta.cl ~ fix(0.00123)

eta.v1 ~ fix(0.133)

add.sd <- 13.7

})

model({

cl <- exp(tcl + eta.cl) * (1000*BWKG)

v1 <- exp(tv1 + eta.v1)

k = cl/v1

d/dt(center) = -k*center

cp=center/v1

IPRED = cp

IPRED ~ add(add.sd)

})

}

Amoxicillin - Keij model

keij_nlmixr_model <- function() {

ini({

tcl <- fix(log(3.22))

tv1 <- fix(log(43))

eta.cl ~ fix(0.267)

eta.v1 ~ fix(0)

prop.sd <- 0.132

add.sd <- 4.48

})

model({

cl <- exp(tcl + eta.cl) * ((BWKG/70)^0.75) * ((PNADAYS/6.8)^0.357) * (((GADAYS/7)/35.8)^2.37)

v1 <- exp(tv1 + eta.v1) * (BWKG/70)

k = cl/v1

d/dt(center) = (-k*center)

cp=center/v1

IPRED = cp

IPRED ~ prop(prop.sd) + add(add.sd)

})

}

Amoxicillin - Tang model

tang_nlmixr_model <- function() {

ini({

tcl <- fix(log(0.81))

tv1 <- fix(log(1.48))

tq <- fix(log(0.17))

tv2 <- fix(log(2.42))

eta.cl ~ fix(0.40)

eta.v1 ~ fix(0)

eta.q ~ fix(0)

eta.v2 ~ fix(0.80)

prop.sd <- 0.35

})

model({

cl <- exp(tcl + eta.cl) * (((BWKG*1000)/3210)^0.75) * (((GADAYS/7)/38.14)^4.19) * ((PNADAYS/7)^0.28)

v1 <- exp(tv1 + eta.v1) * ((BWKG*1000)/3210)

q <- exp(tq + eta.q) * (((BWKG*1000)/3210)^0.75)

v2 <- exp(tv2 + eta.v2) * ((BWKG*1000)/3210)

k = cl/v1

k12 = q/v1

k21 = q/v2

d/dt(center) = (-k*center) + (k21*periph) - (k12*center)

d/dt(periph) = (k12*center) - (k21*periph)

cp=center/v1

IPRED = cp

IPRED ~ prop(prop.sd)

})

}

Benzylpenicillin - Barker model

Barker2023_nlmixr_model <- function() {

# initial conditions/variables are specified

ini({

## Fixed effects

tcl <- log(7.17); label("log Cl (L/h/70kg)") # log of clearance

tv1 <- log(11.8); label("log V1 (L") # log of central compartment

## Random effects

eta.cl ~ 0.249 # IIV variance of clearance

eta.v1 ~ 0 # IIV variance of central compartment

## Residual error

add.err <- 0.241; label("additive error (mg/L)")

prop.err <- 0.012; label("proportional error")

## Covariate effects

bweff.c <- 0.75; label("BWKGeffect on CL")

bweff.v1 <- 1; label("BWKGeffect on V1")

Hill <- 2.68; label("Hill") # Hill coefficient

M <- 0.516; label("M") # fraction of CL on day of birth

N <- 0.020; label("N") # rate of maturation post birth

PMA50 <- 42.6; label("PMA50") # maturation half time (PMA in weeks))

})

# error specification and model specification

model({

cl <- exp(tcl + bweff.c*log(BWKG/70) + Hill*log(PMAWEEKS) -

log(PMA50^Hill + PMAW^Hill) + # maturation function

log(M + (1-M)*(1-exp(-PNA*N))) + # PNA function

eta.cl)

v1 <- exp(tv1 + bweff.v1*log(BWKG/70) + eta.v1)

## ODE

k <- cl/v1

## model differential equation

d/dt(center) = - k * center

## Concentration in plasma

cp <- center / v1 # concentration in plasma

## error model definition

cp ~ prop(prop.err) + add(add.err)

})

}

Benzylpenicillin - Bijleveld model

Bijleveld2018_nlmixr_model <- function() {

# initial conditions/variables are specified

ini({

# Fixed effects

tcl <- log(6.69); label("log CL (L/h/70kg)") # log of clearance

tv1 <- log(41.6); label("log V1 (L)") # log of central compartment

tv2 <- log(70.9); label("log V2 (L") # log of peripheral compartment

tq <- log(0.97); label("log Q (L/h/70kg)")# log of intercompartmental clearance

## Random effects

eta.cl + eta.v1 ~ c(0.45^2,

0.45*0.22*0.84, 0.22^2) # IIV variance-covariance matrix

## Residual error

add.err <- 0.34; label("additive error (mg/L)")

## Covariate effects

bweff.cq <- 0.75; label("BWKGeffect on CL and Q")

bweff.v1v2 <- 1; label("BWKGeffect on V1 and V2")

pnaeff <- 0.37; label("PNADAYSeffect on CL")

tempeff <- 2.58; label("TEMPeffect on CL")

gaeff <- 5.83; label("GADAYSeffect on CL")

})

# error specification and model specification

model({

cl <- exp(tcl + bweff.cq*log(BWKG/70) + pnaeff*log(PNADAYS/2.21) + tempeff*log(TEMP/33.5) + gaeff*log(GADAYS/281) + eta.cl)

v1 <- exp(tv1 + bweff.v1v2*log(BWKG/70) + eta.v1)

v2 <- exp(tv2 + bweff.v1v2*log(BWKG/70))

q <- exp(tq + bweff.cq*log(BWKG/70))

## ODE

k <- cl/v1

k12 <- q/v1

k21 <- q/v2

## model differential equation

d/dt(center) = - k*center + k21*periph - k12*center

d/dt(periph) = k12*center - k21*periph

## Concentration in plasma

cp <- center / v1

## error model definition

cp ~ add(add.err)

})

}

Benzylpenicillin - Muller model

Muller2007_nlmixr_model <- function() {

# initial conditions/variables are specified

ini({

## Fixed effects

tcl <- log(0.103); label("log CL (L/h)") # log of clearance

tv1 <- log(0.359); label("log V1 (L)") # log of central compartment

tv2 <- log(0.152); label("log V2 (L)") # log of peripheral compartment

tq <- log(0.774); label("log Q (L/h)") # log of intercompartmental clearance

## Random effects

eta.cl ~ 0.164 # IIV CL

eta.v1 ~ 0.39 # IIV Vc

## Residual error

add.err <- 1.06; label("additive error (mg/L)")

prop.err <- 0.323; label("proportional error")

})

# error specification and model specification

model({

cl <- exp(tcl + eta.cl)

v1 <- exp(tv1 + eta.v1)

v2 <- exp(tv2)

q <- exp(tq)

## ODE

k <- cl/v1

k12 <- q/v1

k21 <- q/v2

## model differential equation

d/dt(center) = - k*center + k21*periph - k12*center

d/dt(periph) = k12*center - k21*periph

## Concentration in plasma

cp <- center / v1

## error model definition

cp ~ prop(prop.err) + add(add.err)

})

}

Benzylpenicillin - Padari model

Padari2018_nlmixr_model <- function() {

# initial conditions/variables are specified

ini({

## Fixed effects

tcl <- log(13.2); label("log CL (L/h/70kg)") # log of clearance

tv1 <- log(10.3); label("log V1 (L)") # log of central compartment

tv2 <- log(29.8); label("log V2 (L)") # log of peripheral compartment

tq <- log(55.6); label("log Q (L/h/70kg)")# log of intercompartmental clearance

# Random effects

eta.cl + eta.v1 ~ c(0.1521,

0.01, 0.0529) # IIV variance-covariance matrix

eta.v2 ~ 0.1225

eta.q ~ fix(0)

## Residual error

add.err <- 0.278; label("additive error (mg/L)")

prop.err <- 0.13; label("proportional error")

# Covariate effects

bwteff.c <- 0.632; label("BWTeffect on CL")

bwteff.q <- 0.75; label("BWTeffect on Q")

bwteff.v1v2 <- 1; label("BWTeffect on V1 and V2")

Hill <- 3.33; label("Hill") # Hill coefficient

PMA50 <- 55.4; label("PMA50") # maturation half time (PMA in weeks))

})

# error specification and model specification

model({

cl <- exp(tcl + bwteff.c*log(BWKG/70) +

Hill*log(PMAWEEKS) - log(PMA50^Hill +

PMAW^Hill) + # maturation function

eta.cl)

v1 <- exp(tv1 + bwteff.v1v2*log(BWKG/70) + eta.v1)

v2 <- exp(tv2 + bwteff.v1v2*log(BWKG/70) + eta.v2)

q <- exp(tq + bwteff.q*log(BWKG/70) + eta.q)

## ODE

k <- cl/v1

k12 <- q/v1

k21 <- q/v2

## model differential equation

d/dt(center) = - k*center + k21*periph - k12*center

d/dt(periph) = k12*center - k21*periph

## Concentration in plasma

cp <- center / v1

## error model definition

cp ~ prop(prop.err) + add(add.err)

})

}

**References**

1. Australian and New Zealand Neonatal Network. Australasian Neonatal Medicines Formulary. <https://www.anmfonline.org/clinical-resources/> (24 March 2024).

2. Barker CIS, Kipper K, Lonsdale DO et al. The Neonatal and Paediatric Pharmacokinetics of Antimicrobials study (NAPPA): investigating amoxicillin, benzylpenicillin, flucloxacillin and piperacillin pharmacokinetics from birth to adolescence. *J Antimicrob Chemother* 2023; **78**: 2148-61.

3. Nederlands Kenniscentrum voor Farmacotherapie bij Kinderen. Dutch Pediatric Formulary. <https://www.kinderformularium.nl/> (24 March 2024).

4. UpToDate Inc. Pediatric and Neonatal Lexi-Drugs. <https://online.lexi.com/> (24 March 2024).

5. IBM Micromedex. *NeoFax*. Greenwood Village, CO, USA: IBM Watson Health, 2020.

6. Ainsworth S. *Neonatal Formulary: drug use in pregnancy and the first year of life*. Chichester, West Sussex, UK: John Wiley & Sons Inc, 2014.

7. Ministry of Health of Saudi Arabia. *Neonatal Dosage and Practical Guidelines Handbook*, 2014.

8. Swiss Society of Neonatology. Swiss Database for Dosing Medicinal Products in Pediatrics (SwissPedDose). <https://db.swisspeddose.ch/> (24 March 2024).

9. European Committee on Antimicrobial Susceptibility Testing. Clinical breakpoints and dosing of antibiotics. <https://www.eucast.org/clinical_breakpoints> (20 June 2024).

10. Bijleveld YA, Mathôt R, van der Lee JH et al. Population pharmacokinetics of amoxicillin in term neonates undergoing moderate hypothermia. *Clin Pharmacol Ther* 2018; **103**: 458-67.

11. Charles BG, Preechagoon Y, Lee TC et al. Population pharmacokinetics of intravenous amoxicillin in very low birth weight infants. *J Pharm Sci* 1997; **86**: 1288-92.

12. Keij FM, Schouwenburg S, Kornelisse RF et al. Oral and intravenous amoxicillin dosing recommendations in neonates: a pooled population pharmacokinetic study. *Clin Infect Dis* 2023; **77**: 1595-603.

13. Tang BH, Wu YE, Kou C et al. Population pharmacokinetics and dosing optimization of amoxicillin in neonates and young infants. *Antimicrob Agents Chemother* 2019; **63**: e02336-18.

14. Bijleveld YA, de Haan TR, van der Lee JH et al. Evaluation of a system-specific function to describe the pharmacokinetics of benzylpenicillin in term neonates undergoing moderate hypothermia. *Antimicrob Agents Chemother* 2018; **62**: e02311-17.

15. Muller AE, DeJongh J, Bult Y et al. Pharmacokinetics of penicillin G in infants with a gestational age of less than 32 weeks. *Antimicrob Agents Chemother* 2007; **51**: 3720-5.

16. Padari H, Metsvaht T, Germovsek E et al. Pharmacokinetics of penicillin G in preterm and term neonates. *Antimicrob Agents Chemother* 2018; **62**: e02238-17.

17. Jansen SJ, Lopriore E, Bredius RGM et al. Benzylpenicillin serum concentrations in neonates with group B streptococci sepsis or meningitis: a descriptive cohort study. *Pediatr Infect Dis J* 2021; **40**: 434-9.
